# Cannabinoid Receptor 1 Is Required for Neurodevelopment of Striosome-Dendron Bouquets

**DOI:** 10.1523/ENEURO.0318-21.2022

**Published:** 2022-04-08

**Authors:** Jill R. Crittenden, Tomoko Yoshida, Samitha Venu, Ara Mahar, Ann M. Graybiel

**Affiliations:** 1 McGovern Institute for Brain Research, Massachusetts Institute of Technology, Cambridge, MA 02139; 2 Department of Brain and Cognitive Sciences, Massachusetts Institute of Technology, Cambridge, MA 02139

**Keywords:** cannabinoid, CB1R, dopamine, striosome, striosome-dendron bouquet, striatum

## Abstract

Cannabinoid receptor 1 (CB1R) has strong effects on neurogenesis and axon pathfinding in the prenatal brain. Endocannabinoids that activate CB1R are abundant in the early postnatal brain and in mother’s milk, but few studies have investigated their function in newborns. We examined postnatal CB1R expression in the major striatonigral circuit from striosomes of the striatum to the dopamine-containing neurons of the substantia nigra. CB1R enrichment was first detectable between postnatal day (P)5 and P7, and this timing coincided with the formation of “striosome-dendron bouquets,” the elaborate anatomic structures by which striosomal neurons control dopaminergic cell activity through inhibitory synapses. In *Cnr1^−/−^* knock-out mice lacking CB1R expression, striosome-dendron bouquets were markedly disorganized by P11 and at adulthood, suggesting a postnatal pathfinding connectivity function for CB1R in connecting striosomal axons and dopaminergic neurons analogous to CB1R’s prenatal function in other brain regions. Our finding that CB1R plays a major role in postnatal wiring of the striatonigral dopamine-control system, with lasting consequences at least in mice, points to a crucial need to determine whether lactating mothers’ use of CB1R agonists (e.g., in marijuana) or antagonists (e.g., type 2 diabetes therapies) can disrupt brain development in nursing offspring.

## Significance Statement

We report the surprising finding that cannabinoid receptor 1 (CB1R), which mediates the psychoactive effects of marijuana, is required for normal postnatal development of midbrain dopamine-containing neurons and their striatal inputs in mice. We show coincidental onset of CB1R expression in striatal neurons as their axons form bundles with dendrites from dopaminergic neurons. Mice with a mutation in the gene that encodes CB1R show abnormal striatonigral connections and displaced dopamine neurons indicating a postnatal function for CB1R in brain development. These findings raise the need to examine whether disruption of CB1R signaling by therapeutic or recreational CB1R agonists or antagonists use by lactating mothers can permanently impact development of the dopaminergic midbrain in nursing offspring.

## Introduction

Drugs of abuse augment levels of dopamine in striatal forebrain regions and motivate action sequences that can become extreme habits (Nelson and Killcross, 2006; Graybiel, 2008; Everitt, 2014; Gremel and Lovinger, 2017). Among the targets of these drugs are dopamine-containing neurons in the substantia nigra pars compacta (SNc). These neurons give rise to the nigrostriatal tract that degenerates in Parkinson’s disease, and they are vulnerable to abnormality in a range of motor and neuropsychiatric disorders. The nigrostriatal tract is known in the normal state to modulate not only movement but also psychic vigor, motivation and reinforcement-based learning.

Within the striatum, specialized widely distributed zones known as striosomes (also known as patches) respond to psychomotor stimulants such as amphetamine by increased expression of immediate-early response genes (Graybiel et al., 1990; Moratalla et al., 1996; Canales and Graybiel, 2000; Jedynak et al., 2012; Crittenden et al., 2017, 2021a; Prager and Plotkin, 2019). Axons from projection neurons in striosomes are enriched in cannabinoid receptor 1 (CB1R) and innervate clusters of dopamine-containing neurons in the ventral tier of the SNc (SNcv) to form anatomically conspicuous “striosome-dendron bouquets” (Davis et al., 2018). Striosomal axons innervate the SNcv and also intertwine themselves in synapse-rich bundles of dopamine-containing dendrites (dendrons) that protrude deep into the substantia nigra pars reticulata (SNr), a major basal ganglia output nucleus (Crittenden et al., 2016). It now has been shown that optogenetic stimulation of striosomal axons within striosome-dendron bouquets can fully inhibit spike activity of dopamine-containing neurons (Lee and Tepper, 2009; McGregor et al., 2019; Evans et al., 2020) and further can produce rebound activation of the dopamine neurons (Evans et al., 2020).

Endocannabinoids as well as therapeutic and recreational cannabinoids bind to the receptors CB1R and CB2R (Ashton et al., 2008). CB1R is thought to mediate most of the psychoactive and habit-forming qualities of marijuana (Zimmer et al., 1999; Covey et al., 2017; but see Liu et al., 2017). Together, this evidence strongly suggests that CB1Rs, along with other receptors expressed in the bouquets such as the μ-opioid receptor (MOR1; Crittenden et al., 2016), could have marked effects on neural circuit function, behavior and addiction by controlling the activity of dopamine-containing neurons (Riegel and Lupica, 2004; Davis et al., 2018). Functions of striosome-dendron bouquets in adults, however, are yet to be reported.

Here, we demonstrate that striosome-dendron bouquets form in the early postnatal period, coinciding with the enrichment of CB1R in striosomal neurons and their axons. Moreover, mice lacking CB1R expression have abnormal placement of striosomal axons and dopaminergic dendrites such that the bouquets appear disorganized already in the early postnatal period and in adulthood, indicating that CB1R signaling in the postnatal brain controls the wiring of the striosome-nigral circuit.

## Materials and Methods

### Mice

All animal procedures were approved by the Committee on Animal Care at Massachusetts Institute of Technology (MIT), which is AAALAC accredited. Frozen embryonic heterozygous *Cnr1^−/+^* mice (B6.129-Cnr1<tm1Zim>/Ieg; #EM:02,274, RRID: 3795243), created by A. Zimmer (Zimmer et al., 1999), were imported from the European Mouse Mutant Archive (EMMA) to MIT to establish a colony. Mice carrying the *P172-mCitrine* striosomal transgene marker (Piggyback-Tta, Line P172 C57B6J, http://enhancertrap.bio.brandeis.edu/data/; Shima et al., 2016) were imported to MIT to establish a colony as previously described (Crittenden et al., 2016). Knock-out (KO) and sibling control pups were generated from pairs of *Cnr1^−/+^* heterozygous mice. In cases where the *P172-mCitrine* transgene was used, *Cnr1^−/+^* heterozygous mice were crossed to *P172-mCitrine* hemizygous mice, and *Cnr1^−/+^* offsprings carrying the *P172-mCitrine* transgene were then crossed to *Cnr1^−/+^* mice to generate *Cnr1^−/−^* KOs and *Cnr1^+/+^* controls that each carry one copy of the *P172-mCitrine* transgene. Mice were genotyped by Transnetyx for *neomycin* (present at the *Cnr1* deletion site), the wild-type *Cnr1* allele, and *TRE* (*tetracycline-responsive element* present at the *P172-mCitrine* transgene site).

Mice were group-housed, had free access to food and water and were maintained on a 12/12 h light/dark cycle (lights on at 7:00 A.M.). Roughly equal numbers of each sex were used for all experiments. Mice imaged for *P172-mCitrine* expression were evaluated at 5.5 weeks of age or younger because of age-dependent silencing of the *P172-mCitrine* transgene (Shima et al., 2016). Exceptions were 70-d-old *P172-mCitrine* mice that were used for circuit tracing, as described in the Results. *Cnr1^−/−^* KO mice of both sexes were compared and no gross differences between sexes were evident for the described brain phenotype.

### Tissue preparation and immunoreactions

For a detailed protocol, see Crittenden et al. (2021b). For brain collection from adults, mice were deeply anesthetized with Euthasol (pentobarbital sodium and phenytoin sodium from Virbac AH Inc.) before transcardial perfusion with 20 ml of 0.9% saline and 60 ml of freshly depolymerized 4% paraformaldehyde in 0.1 m NaKPO_4_ buffer. Brains were then dissected, postfixed for 90 min and stored in 25% glycerol sinking solution overnight or until cutting. For whole mount preparations, mice were perfused with heparin before fixative to eliminate fluorescence from red blood cells.

For brain tissue collection from pups, neonatal mice [postnatal day (P)0–P5] were anesthetized on ice and older pups (P7, P11) by isoflurane inhalation exposure. After removing the scalp, the whole head was submerged in freshly depolymerized 4% paraformaldehyde in 0.1 m NaKPO_4_ buffer and placed on a low-speed shaker for 3 d at 4°C. The tissue was then transferred to a 25% glycerol solution and shaken overnight. Brain tissue was then dissected from the skull and returned to 25% glycerin until cutting.

To prepare sections, brains were frozen on dry ice to generate 30-μm (adult samples) or 60-μm (pup samples) free-floating coronal sections with a freezing microtome. Sections were stored in 0.1% sodium azide in 0.1 m NaKPO_4_ solution.

For the immunoreactions, sections were rinsed three times for 5 min each with 10 mm NaPO_4_, 150 mm NaCl, and 2.7 mm KCl with 0.2% Triton X-100, and then blocked by shaking for 60 min in TSA Blocking reagent (PerkinElmer). Sections were left shaking for one to three nights at 4°C in primary antibodies. Primary antibodies and concentrations used were: goat anti-CB1R (Frontier Institute CB1-Go-Af450, RRID: AB_2571592), 1:100; rat anti-DAT (Millipore MAB369, RRID: AB_2190413), 1:200; rabbit anti-TH (Abcam ab112, RRID: AB_297840), 1:4000; chicken anti-GFP (to detect mCitrine; Abcam ab13970, RRID: AB_300798), 1:2000; rabbit anti-MOR1; Abcam Ab134054), 1:500; rabbit anti-FoxP2 (Sigma-Aldrich HPA-000382, RRID: AB_1078908), 1:1000. After rinsing, secondaries were applied at a 1:300 dilution for an overnight reaction at 4°C with gentle shaking. Secondary antibodies were: anti-goat Alexa Fluor 647 (Thermo Fisher Scientific A21447, RRID: AB_2535864), anti-rat Alexa Fluor 546 (Thermo Fisher Scientific A11081, RRID: AB_2534125), anti-rabbit Alexa Fluor 546 (Thermo Fisher Scientific A10040, RRID: AB_2534016), anti-chicken FITC (Abcam ab63507, RRID: AB_1139472). Sections were mounted on subbed glass slides and coverslipped using ProLong Antifade Reagent with DAPI (Thermo Fisher Scientific).

### Whole-brain clearing

Perfused brains were bisected and trimmed to contain mainly the basal ganglia. Brains were cleared using the uDISCO method (Pan et al., 2016) as follows. Samples were dehydrated by gentle shaking in increasing concentrations of *tert*-butanol in dH_2_O at room temperature: 30% for 4 h, 50% for 4 h, 70% overnight, 80% for 4 h, 90% for 4 h, and 96% overnight. Samples were then incubated in dichloromethane for 50 min and moved into a solution of BABB-D10 [1:2 ratio of benzyl alcohol and benzyl benzoate (BABB)], 10:1 ratio of BABB and diphenyl ether, 0.4% vol vitamin E for overnight incubation and further storage until imaging. All incubation steps were performed at room temperature under a fume hood. For confocal imaging, samples were transferred into BABB-D10 filled chambers with 1.5 coverslip-equivalent bottoms (ibidi Inc.).

### Microscopy

For fluorescence microscopy, a Zeiss AxioZoom microscope was used to obtain wide-field images with standard epifluorescence filter sets for DAPI (365 excitation, 395 beamsplitter, 445/50 emission), eGFP/AF488 (470/40 excitation, 495 beam splitter, 525/50 emission), tdTomato/AF546 (550/25 excitation, 570 beamsplitter, 605/70 emission) and Cy5/AF647 (640/30 excitation, 660 beamsplitter, 690/50 emission). Confocal imaging on sections and whole mount tissue was performed with a Zeiss LSM710 with diode lasers (473, 559, 653 nm) for excitation. Images were collected with a 10× 0.4 NA objective and a 60× oil 1.3 NA objective lens. Optical sectioning was optimized according to the microscope software. Images were processed and analyzed with Fiji software (Schindelin et al., 2012; RRID: SCR_002285) or, for 3D reconstruction and movies, Imaris 9.5 software (Oxford Instruments; RRID: SCR_007370). Figures were prepared with Photoshop 22.4.2 and Illustrator 6.0 (Adobe).

### Histologic measurements

For striosomal counts, for striosomal and matrix compartment area calculations, and for average MOR1 immunointensity calculations in striosomes and matrix, measurements were made from images acquired on a Zeiss AxioZoom microscope. Each image was converted into 8-bit and analyzed with Fiji software. Fluorescence intensities were normalized by subtracting background fluorescence for each sample. A genotype blind investigator used the freehand selection tool to draw the boundaries of striosomes using MOR1 immunolabeling. Any striosomes that appeared connected were counted as one unit. Mean intensity value and pixel area were measured for each striosome and for the whole surrounding matrix.

Striosome-dendron bouquet and SNcv size measurements were made from images acquired on a Zeiss AxioZoom microscope and analyzed using Photoshop 23.1 (Adobe) and Fiji software. Each image was first converted into 8-bit, and fluorescence intensities were normalized by subtracting background fluorescence for each sample. Thresholding was used to create defined borders for dendrons. Measurements were made by investigators blinded to genotype. Using both the dopamine transporter (DAT) and MOR1 channel, dendron widths (medial-lateral aspect) were measured using the ruler tool at two different distances, 50 and 100 μm, ventral to the SNcv/SNr border. SNcv regions were defined as double-labeled for DAT and MOR1, and thicknesses (dorsal-ventral aspect) were measured at sites adjacent to dendrons.

### Cell counting

Coronal brain sections at the anterior, mid-level and posterior striatum were labeled with DAPI and immunolabeled for P172-mCitrine and FoxP2, and then imaged on a Zeiss AxioZoom microscope. Sections from *Cnr1^−/−^* KO and sibling control mice were age-matched (27- to 33-d-old range) and samples from each sex were taken and processed in the same way and in parallel by individuals blinded to genotype. In images of sections immunolabeled for MOR1, the striatum and striosomes were outlined by hand, and P172-mCitrine-positive and FoxP2-positive cells within striosomes of the dorsal striatum (dorsal to the anterior commissure) were counted in each section. The entire area of the striatum was measured to calculate the overall density of P172-mCitrine-positive and FoxP2-positive striosomal neurons.

Identification of striosomes and immunopositive cells was assisted by Fiji software. The images were converted to 16-bit gray levels and inverted. The dorsal striatal area was selected, background signal was subtracted using the “Rolling Ball” command (radius = 500 μm), the “Analyze Particles” command was applied and verified by eye to be identifying bona fide cells, and the P172-mCitrine-positive and FoxP2-positive cell counts were recorded.

### Statistics

Histologic data measurements from multiple sections for each adult mouse (three striatal and six nigral hemisphere sections per mouse) were averaged to obtain one value per hemisphere per mouse. Each animal’s average value per hemisphere was plotted and used for SD calculations to reflect inter-animal variability. Statistical differences between groups were assessed by unpaired, two-tailed Student’s *t* tests. Specific *p* values are given when *p *<* *0.05 and considered statistically significant.

## Results

### Striosome-dendron bouquets in the substantia nigra appeared severely disorganized in *Cnr1^−/−^* KO mice

As a first test of CB1R function in striosomes and their axonal projections to bouquets, we examined brains of *Cnr1^−/−^* KO mice (Zimmer et al., 1999) that were crossed to transgenic mice carrying a striosome-enriched fluorophore marker, P172-mCitrine (Crittenden et al., 2016; Shima et al., 2016). *P172-mCitrine* transgenic mice were generated as part of a project in which the PiggyBac transposon was employed to insert the fluorophore-encoding *mCitrine* transgene at many different loci throughout the genome to yield mouse lines that label a variety of neuronal cell types. The *P172-mCitrine* line was discovered to drive highly specific expression in striosomal projection neurons that target striosome-dendron bouquets (Crittenden et al., 2016; Shima et al., 2016).

**Figure 1.**
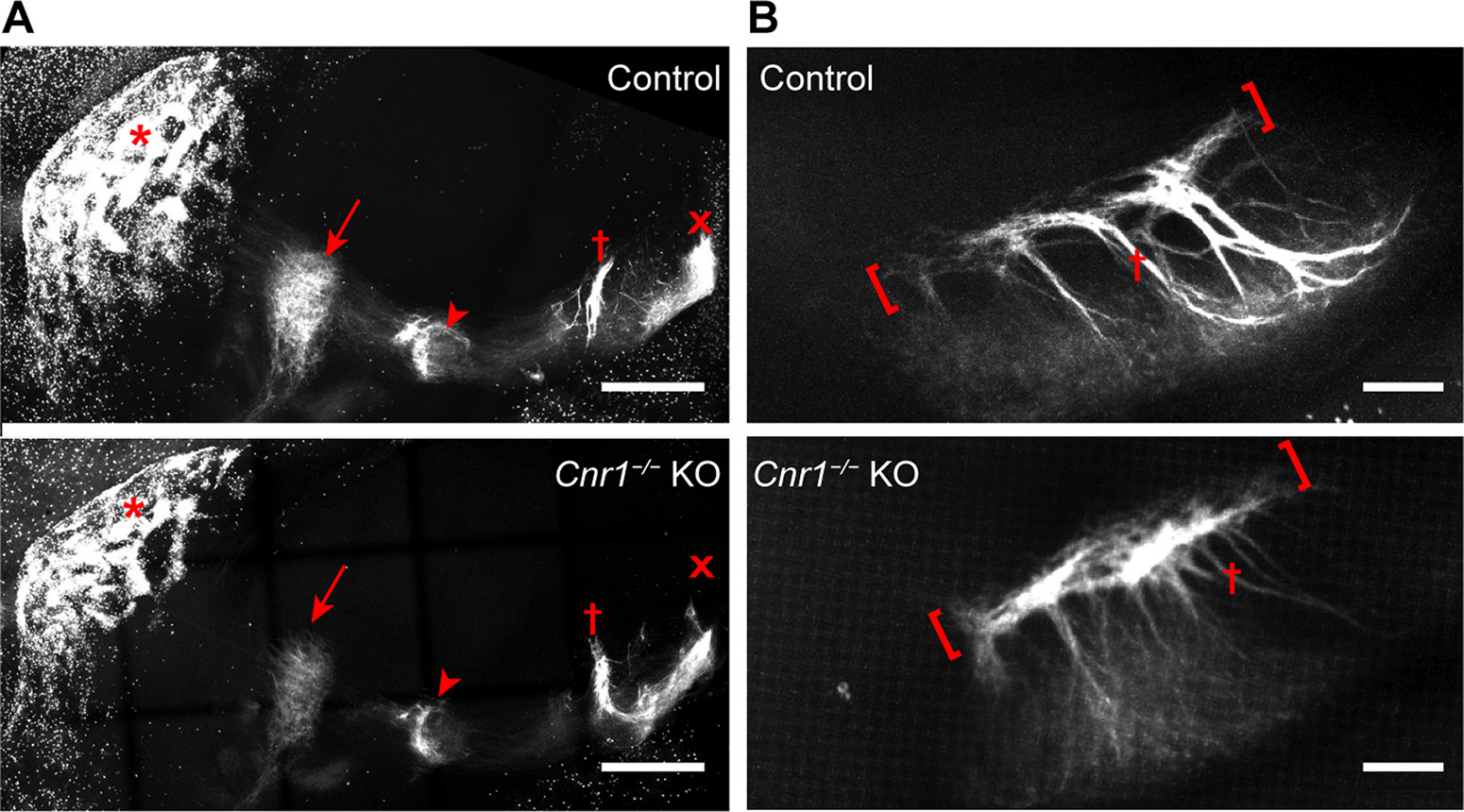
Striosomal axons reach their targets but striosome-dendron bouquets are severely malformed in the SN of *Cnr1^−/−^* KO mice. To visualize the entire striosome-nigral projection, confocal imaging was done on whole cleared brains from juvenile (∼P30) control and sibling *Cnr1^−/−^* KO mice carrying the striosomal fluorescent marker P172-mCitrine (shown in white). ***A***, Sagittal maximum-projection images show striosomal neurons clustered in patches (examples at red asterisks) within the striatum. Striosomal axon projections are visible in the external and internal globus pallidus (arrow and arrowhead, respectively) and the rostral and caudal portions of the substantia nigra (red crosses indicate location of bouquets and red x indicates location of posterior dopamine cell cluster regions). Anterior is to the left. ***B***, Maximum-projection coronal view of the rostral nigra and all of its bouquets made visible by P172-mCitrine fluorescence in striosomal axons. In *Cnr1^−/−^* KO mice compared with controls, “stems” of bouquets (examples designated by red crosses) appear thinner whereas the SNcv/SNr border (red-bracketed regions) appear thicker. Medial is to the left. Scale bars: 400 μm (***A***) and 100 μm (***B***). See corresponding 3D Movies 1, 2.

We imaged the entire basal ganglia in cleared whole brains from *Cnr1^−/−^* KO and control mice carrying the *P172-mCitrine* transgene, using confocal fluorescence imaging and 3D reconstruction (Fig. 1). Similar to their sibling control mice, *Cnr1^−/−^* KO mice had striosomes with axons that project toward their intended nigral target in the midbrain (Fig. 1*A*). However, within the SN there were severe abnormalities in the distribution of striosomal fibers (Fig. 1*B*). Instead of forming discrete striosome-dendron bouquet “stems” as in the controls, the P172-mCitrine striosomal fibers in *Cnr1^−/−^* KO mice appeared in a thick pile-up at the presumed SNcv/SNr border where dopaminergic cell bodies of the SN ventral tier reside (Crittenden et al., 2016). The appearance of striosome-dendron bouquets in brain sections depends on the level and plane of section. To ensure that our impression of bouquet differences between *Cnr1^−/−^* KOs and controls was not simply a reflection of this variability, we used confocal optical sectioning and 3D reconstruction from blocks of tissue that contained the entire basal ganglia and created 3D movies of striosomal neurons and their projections. These movies show that striosomal bouquet fibers were indeed grossly disorganized in *Cnr1^−/−^* KO mice (Movies 1, 2).

Movie 1. 3D visualization of striosomal neurons and projections in the basal ganglia of adult control mice that carry the *P172-mCitrine* striosomal marker transgene. Striosomal neurons and their projections are visualized by direct fluorescence for *P172-mCitrine* expression, shown in white. The movie starts with a sagittal view of the entire basal ganglia within one hemisphere, with anterior to the left as shown in Figure 1*A*. The images are rotated counterclockwise to first focus on the frontal aspect of the striatum with clusters of striosomal neurons in white, followed by a zoom-in to focus on bouquets in the SN of the midbrain. Note the sharp SNcv/SNr border and robust dorsoventrally oriented bouquet stems in controls as compared to a less distinct SNcv border and thinner bouquet stems in *Cnr1^−/−^* KO mice (Movie 2). 10.1523/ENEURO.0318-21.2022.video.1

Movie 2. 3D visualization of striosomal neurons and projections in the basal ganglia of adult *Cnr1^−/−^* KO mice that carry the *P172-mCitrine* striosomal marker transgene. Striosomal neurons and their projections are visualized by direct fluorescence for *P172-mCitrine* expression, shown in white. The movie starts with a sagittal view of the entire basal ganglia within one hemisphere, with anterior to the left as shown in Figure 1*A*. The images are rotated counterclockwise to first focus on the frontal aspect of the striatum with clusters of striosomal neurons in white, followed by a zoom-in to focus on bouquets in the SN of the midbrain. Note the less distinct SNcv/SNr border and thinner dorsoventrally oriented bouquet stems in *Cnr1^−/−^* KO mice relative to the sharp SNcv border and robust bouquet stems in controls (Movie 1). 10.1523/ENEURO.0318-21.2022.video.2

### Striosomal and dopaminergic neuropil is disorganized at the SNcv/SNr border

**Figure 2.**
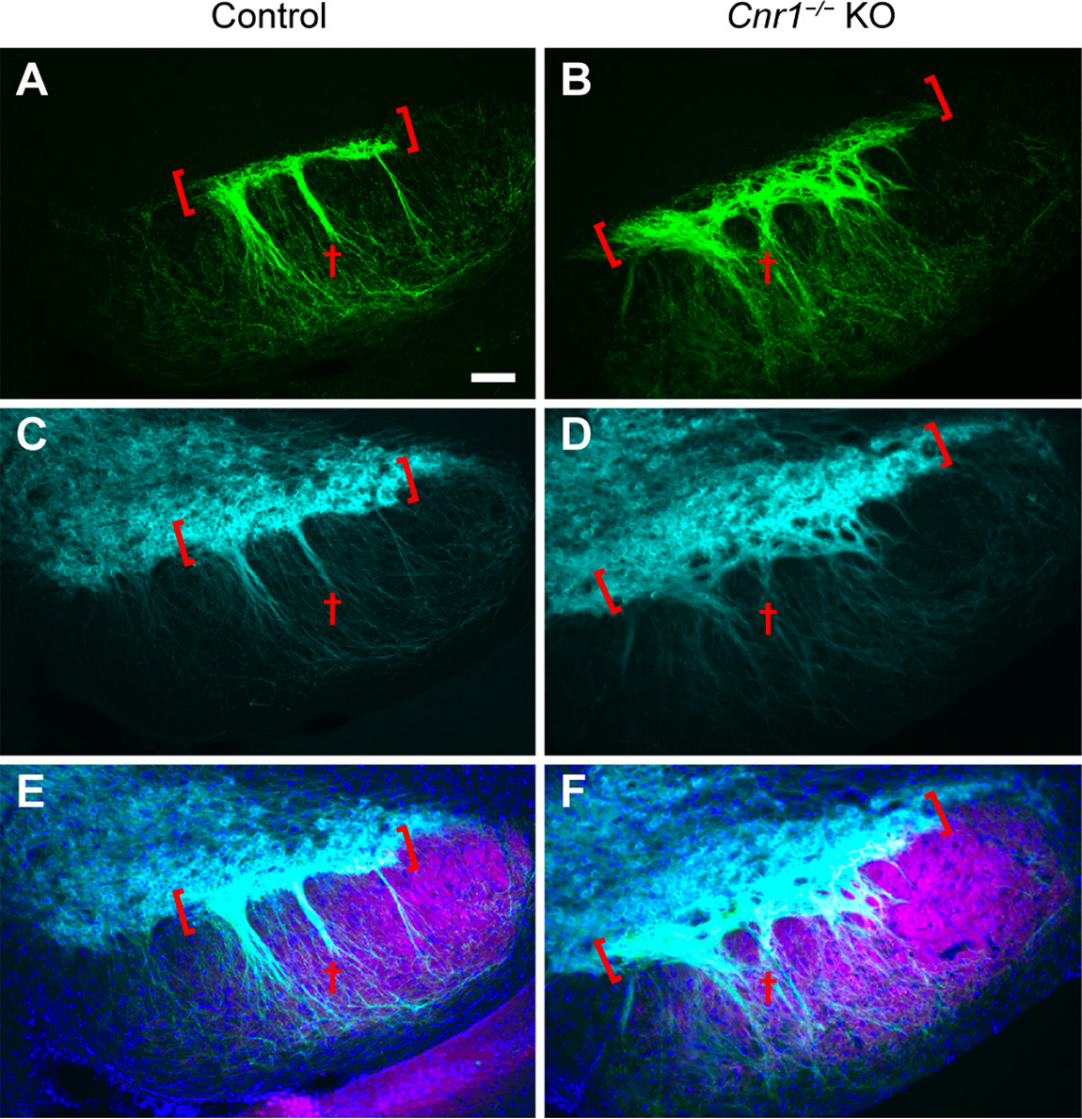
Striosomal axons and dopaminergic dendrites are disorganized in the SN of *Cnr1^−/−^* KO mice. Sections through the SN (left hemispheres) immunolabeled for the striosomal marker P172-mCitrine (***A***, ***B***), the dopaminergic cell marker DAT (***C***, ***D***) and merged with an SNr marker in magenta (***E***, ***F***). In *Cnr1^−/−^* KO mice relative to controls, both striosomal and DAT-positive neuropil appear unorganized and looser, failing to define either a sharp SNcv/SNr border (red brackets) or discrete bundles of striosomal axons and dopamine dendrites bundles in the SNr (red crosses). Scale bar: 100 μm (applies to all panels). *N* > 3 for each genotype. See Extended Data Figure 2-1*A*,*B* for more samples.

To confirm that the bunched striosomal fibers in *Cnr1^−/−^* KO mice were indeed located at the SNcv/SNr border, we co-immunolabeled individual brain sections for DAT to allow detection of dopamine-containing neurons and their processes. In control mice, the dopamine-containing cell bodies of the SNcv formed a tight layer with a sharp border between them and the SNr neuropil (Fig. 2*A*,*C*,*E*). In sharp contrast, *Cnr1^−/−^* KO mice had a markedly less discrete SNcv/SNr border and apparent disorganization of both dopaminergic dendrites (DAT-positive) and striosomal axons (P172-mCitrine-positive; Fig. 2*B*,*D*,*F*; Extended Data Fig. 2-1*A*,*B*). Although the overall distribution of dopaminergic neurons was reported to be normal in *Cnr1^−/−^* KO mice (Steiner et al., 1999; Gargano et al., 2020), our new findings show that their fine-scale dendritic arbors, as well as their input circuits, were severely disrupted.

10.1523/ENEURO.0318-21.2022.f2-1 Extended Data Figure 2-1 Striosome-dendron bouquet samples in in the SN of controls (***A***) and *Cnr1^−/−^* KO (***B***) mice. Images of the SN from three mice of each genotype showing disorganized and loosely fasciculated striosomal axons (P172-mCitrine fluorescence in green) and dopaminergic dendrites (cyan, DAT immunolabeling in cyan). Coronal sections from the left hemisphere are shown. Scale bars: 100 μm. Download Figure 2-1, TIF file.

**Figure 3.**
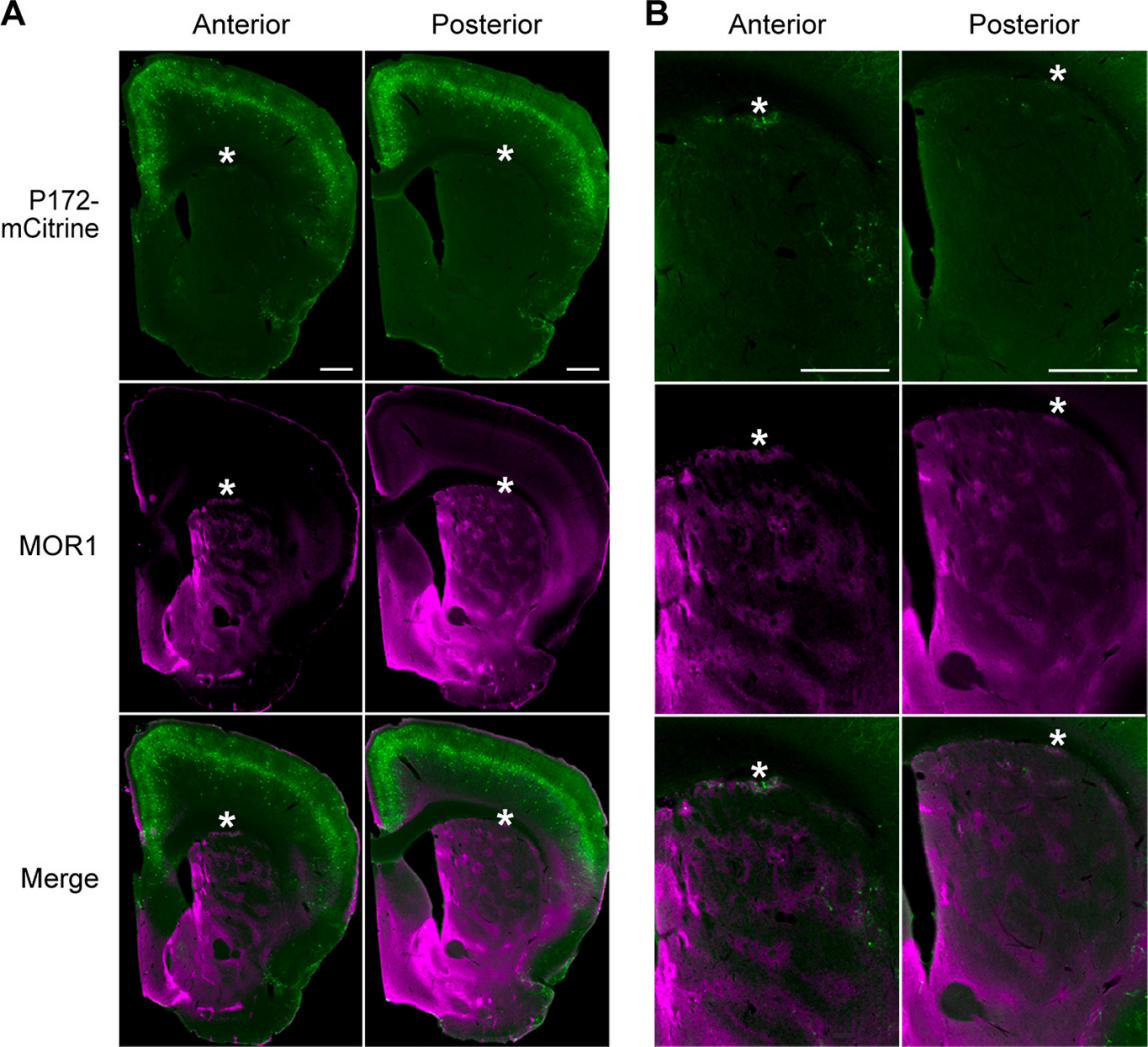
Sparse striosomal cell labeling in aged P172-mCitrine transgenic mice. ***A***, By P172-mCitrine fluorescence (green), many cells are visible in the cerebral cortex, but only a few cells remain labeled in the striosomes (identified by MOR1 labeling in magenta) of transgenic mice at 70 d of age. ***B***, Higher magnification view of sections in ***A*** showing P172-mCitrine-positive neurons in striosomes in the striatum. Coronal hemispheric sections through the anterior (top row) and posterior (bottom row) striatum are shown. Medial is to the left. Scale bars: 500 μm (***A***, ***B***, top panels). Similar results in *N *=* *3 mice aged 70 d. Asterisks are inserted above examples of striosomes located within the subcallosal streak.

**Figure 4.**
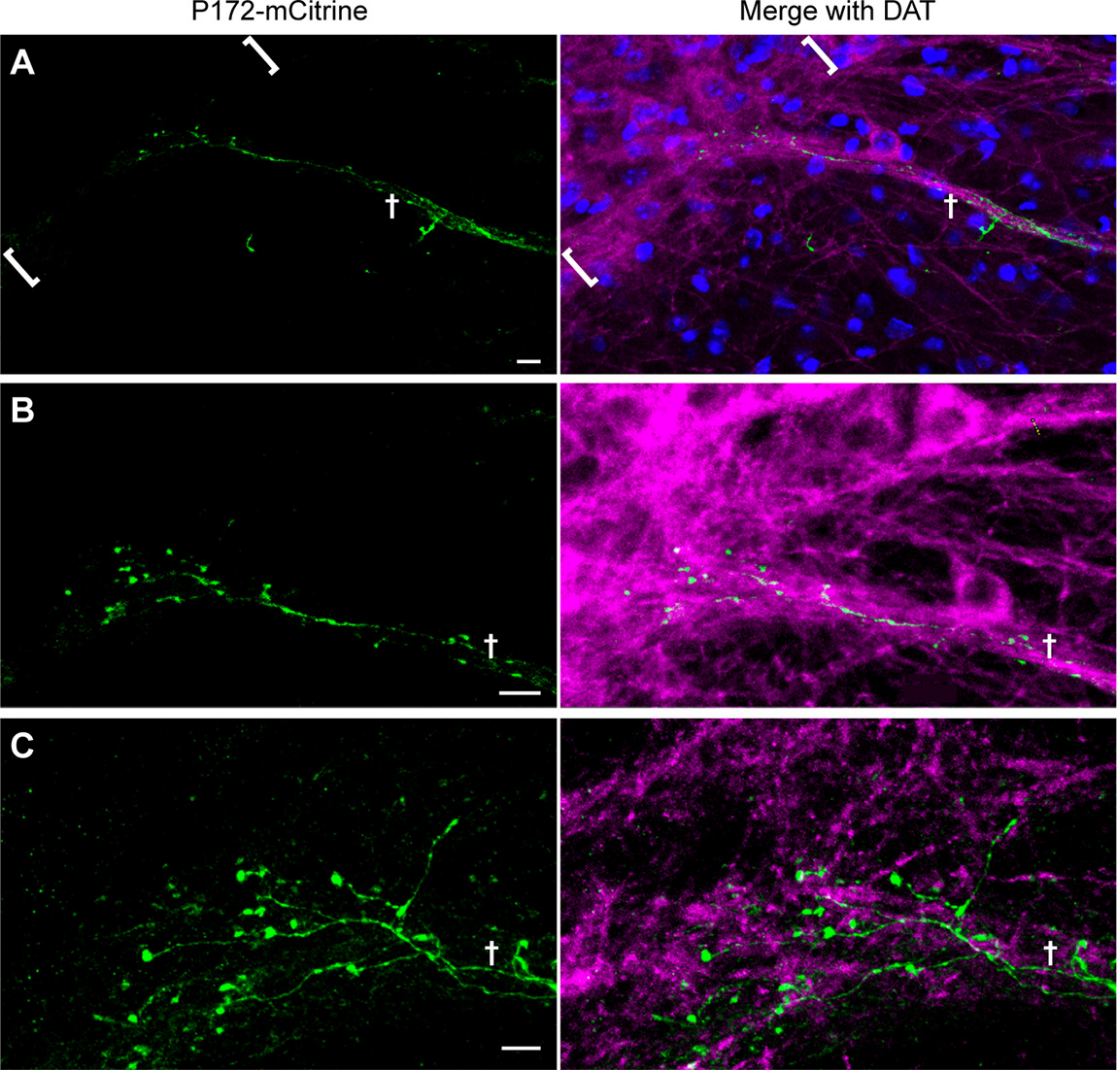
Sparse striosomal axon labeling in bouquets of aged P172-mCitrine transgenic mice. Increasingly magnified images (top to bottom) show what appears to be a single P172-mCitrine-positive (green) striosomal fiber climbing a bundle of dopaminergic dendrites labeled for DAT (magenta) and branching to form multiple end bulbs in the SNcv bordering the SNr. DAPI labels all cell nuclei in blue. The SNcv is delineated by brackets in ***A***, and the dendron is designated by a cross in ***A–C***. Scale bars: 10 μm (***A***, ***B***) and 5 μm (***C***). Maximum projection through a total of 18 0.5-μm sections. Similar results in *N *=* *3 mice aged 70 d. Nigral hemispheric sections are shown with medial aspect to the left.

*P172-mCitrine* transgene expression in the striatum was previously noted to be diminished in the striatum by 5.5 weeks of age (Crittenden et al., 2016), presumably owing to developmental changes in the enhancers that control transgene expression (Shima et al., 2016). This age-dependent loss of *P172-mCitrine* expression raised the importance of using age-matched or sibling mice to compare expression in *Cnr1^−/−^* KO mice and controls, as described in the Methods. We nevertheless capitalized on the striosome-selective loss of expression to bolster the evidence that the P172-mCitrine-labeled fibers in bouquets arise from striosomal neurons. This is particularly relevant here because some cortical neurons exhibit labeling in *P172-mCitrine* transgenic mice, and cortical neurons have axon pathfinding defects in *Cnr1^−/−^* KO mice (Wu et al., 2010; Alpár et al., 2014; Saez et al., 2020). We reasoned that if there were an age-dependent loss of labeling in striosomal neurons in *P172-mCitrine* mice, then we should see a corresponding loss of fiber labeling in bouquets. Indeed, in 70-d-old *P172-mCitrine* mice that we examined, P172-mCitrine labeling was maintained in the cerebral cortex but only a few neurons remained labeled in the striosomes (Fig. 3*A*,*B*), along with only a few remaining fibers labeled in striosome-dendron bouquets. The individually labeled striosomal axons appeared to have varicosities that were in contact with bundled dopaminergic fibers descending into the SNr as well as axon end bulbs around dopaminergic fibers and neurons in the SNcv, bordering the SNr (Fig. 4*A–C*). These results support our conclusion that the disorganized bouquet fibers in *Cnr1^−/−^* KO mice arise from neurons within striosomes, which are normally enriched for CB1R expression (Davis et al., 2018).

**Figure 5.**
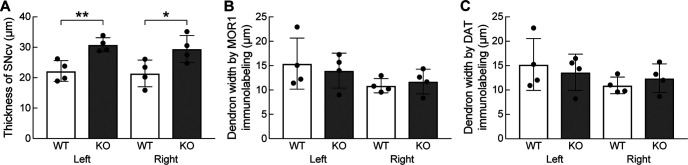
Abnormally thick SNcv in adult *Cnr1^−/−^* KO mice. ***A***, The average dorsoventral thickness of the SNcv border, defined as immunopositive for both MOR1 and DAT, was significantly greater as measured in coronal nigral sections from *Cnr1^−/−^* KOs than in controls (**p =* *0.0076 for left hemisphere and *p =* *0.042 for right hemisphere). ***B***, ***C***, The averaged mediolateral width of the dendron, measured by either MOR1 or DAT labeling at 50 and 100 μm ventral to the SNcv/SNr border, was not significantly different between controls and *Cnr1^−/−^* KOs (*p *=* *0.70 and *p *=* *0.85 for MOR1 and *p *=* *0.64 and *p *=* *0.43 for DAT for left and right hemispheres, respectively). Left and right designate opposite hemispheres from the same mice. Means for each animal and SDs of interanimal variability are plotted. Images from which measurements were made are shown in Extended Data Figure 5-1.

Relative to the P172-mCitrine marker, MOR1 is stably expressed in striosomes as mice age, although it is not as strongly and discreetly expressed in striosomal bouquet axons (Crittenden et al., 2016). For genotype comparison of the bouquet phenotype in fully adult mice, we co-labeled sections for DAT and MOR1. We measured both the dorsal-ventral thickness of the SNcv/SNr border region and of the bouquet stem width in brain sections that contained bouquets from *Cnr1^−/−^* KO and control mice. Bilateral measures were made by a person blind to genotype. We found that the SNcv/SNr border region, as defined by co-labeling for MOR1 and DAT, was significantly thicker in *Cnr1^−/−^* KO mice than in controls (*p *=* *0.0076, left hemisphere; *p *=* *0.042, right hemisphere by unpaired Student’s *t* test, *N *=* *4 mice per genotype, equally divided and matched for sex, *n *=* *6 hemisphere slices per mouse; Fig. 5*A*; Extended Data Fig. 5-1). This finding is consistent with what we observed by eye in single sections and in whole-nigral movies labeled for the striosomal marker P172-mCitrine (Figs. 1*B*, 2; Movies 1, 2; Extended Data Fig. 2-1). However, the widths of the bouquet stems were not significantly different between genotypes based on MOR1 and DAT labeling (*p *=* *0.70 and *p *=* *0.85 for MOR1 and *p *=* *0.64 and *p *=* *0.43 for DAT for left and right hemispheres, respectively, by unpaired Student’s *t* test, *N *=* *4 mice per genotype, equally divided and matched for sex, *n *=* *6 hemisphere slices per mouse; Fig. 5*B*,*C*). For technical reasons, we did not examine their taper.

10.1523/ENEURO.0318-21.2022.f5-1 Extended Data Figure 5-1 A subset of images from coronal nigral sections used to measure anatomical features in adult *Cnr1^−/−^* KO mice and controls. The SNcv were defined for being immunopositive for both MOR1 and DAT and are delineated by red brackets in the merged image; dendrons are designated by a red cross. Measurements were taken along the white line (one line per section). *N *=* *4 mice of each genotype, balanced for sex, *n *=* *3 sections per hemisphere for each mouse. Scale bar: 10 μm (lower right panel and applies to all panels). Download Figure 5-1, TIF file.

### There were few significant differences in MOR1 and DAT immunolabeling intensities in *Cnr1^−/−^* KO mice versus controls

We also compared the MOR1 and DAT markers in *Cnr1^−/−^* KO mice and controls according to their immunointensity. We measured MOR1 and DAT intensity within the SNcv and within dendrons in the regions used for measuring SNcv thickness and dendron width. No significant genotype differences were found (by unpaired Student’s *t* test, *N *=* *4 mice per genotype, equally divided and matched for sex, *n *=* *3 left hemisphere slices per mouse*, p *>* *0.05 for all comparisons between genotypes except for MOR1 immunointensity in left hemisphere dendrons for which *p *=* *0.046; Extended Data Fig. 5-2*A–D*). The trend for less MOR1 and DAT immunolabeling of dendrons in the left hemisphere of *Cnr1^−/−^* KO mice (Extended Data Fig. 5-2*C*,*D*) could be related to the tendency for sparser dendrons as seen in some samples (Figs. 1*B*, 2*B*,*D*,*F*; Movie 2; Extended Data Fig. 2-1*B*). The finding that DAT and MOR1 immunointensity in the SNcv was not different from controls is important to support that the thickened SNcv phenotype in *Cnr1^−/−^* KO mice is not an artifact of stronger fiber labeling.

10.1523/ENEURO.0318-21.2022.f5-2 Extended Data Figure 5-2 Immunolabeling for MOR1 and DAT in nigral sections from *Cnr1^−/−^* KO mice and controls. There were few significant genotype differences in MOR1 (***A***, ***C***) and DAT (***B***, ***D***) immunolabeling (arbitrary units) of SNcv (***A***, ***B***) or dendrons (***C***, ***D***). Genotype comparisons were made only between same-side hemispheres because they were processed together but separately from the opposite hemisphere (*p *>* *0.05 for comparisons between genotypes except for MOR1 immunointensity in left hemisphere dendrons for which *p =* *0.046). Download Figure 5-2, TIF file.

**Figure 6.**
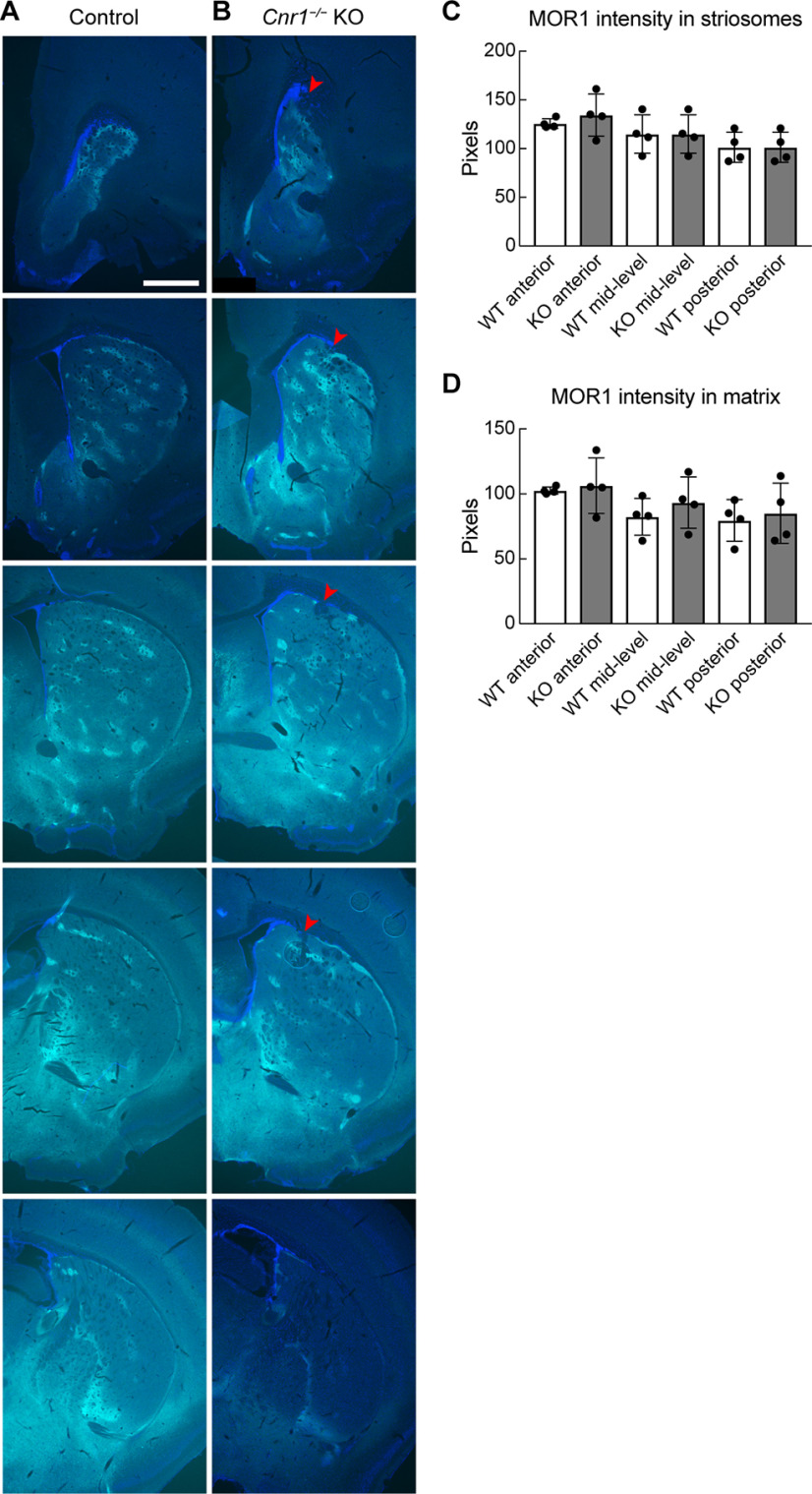
MOR1 striosomal labeling in adult *Cnr1^−/−^* KO mice. ***A***, ***B***, The expression level and pattern of labeling for the striosomal immunomarker MOR1 (cyan) are similar in serial sections from a control (***A***) and a *Cnr1^−/−^* KO (***B***) mouse. The left striatal hemisphere is shown from anterior (top) to posterior (bottom). Cell nuclei are labeled by DAPI (blue). Red arrowhead in *Cnr1^−/−^* KO panels indicates the abnormally bunched corticofugal fibers near the border of the dorsolateral striatum. Scale bar: 500 μm (upper left panel and applies to all panels). *N *=* *4 mice imaged for each genotype. ***C***, ***D***, Average MOR1 immunointensities (arbitrary units) in striosomes and matrix compared between genotypes at three anteroposterior levels. The middle three sections shown in ***A***, ***B*** are representative of the levels chosen. No significant genotype differences were found (*p *>* *0.05 for all comparisons). Means for each animal and SDs of interanimal variability are plotted.

Tests for changes in MOR1 expression in the striatum are particularly relevant for *Cnr1^−/−^* KO mice, which we report here have anatomic abnormalities in their SNcv dopamine systems, because of previous findings indicating (1) that disruption of SNcv dopaminergic neuropil leads to reduced MOR1 immunolabeling in striata of rodents (Johansson et al., 2001) as a possible compensatory response to increased levels of the MOR ligand enkephalin detected by immunolabeling (Koizumi et al., 2013) and (2) that enkephalin itself is increased in the striata of *Cnr1^−/−^* KO mice (Steiner et al., 1999). We measured MOR1 immunoreactivity within striosome and matrix compartments of the striatum at three anterior-posterior levels (Fig. 6*A*,*B*) but found no genotype differences between *Cnr1^−/−^* KO mice and controls (*p *>* *0.05 for all comparisons between genotypes by unpaired Student’s *t* test, *N *=* *4 mice per genotype, equally divided and matched for sex, *n *=* *3 hemisphere slices per mouse at matched levels; Fig. 6*C*,*D*).

### Striosomal areas and neuronal counts were not significantly different in *Cnr1^−/−^* KO mice versus controls

To determine whether the terminal field abnormalities of the striosome-nigral axons reflected a distortion of striatal striosome organization, we examined striatal anatomy with the striosomal neuropil marker MOR1 and the striosomal cell body markers P172-mCitrine and FoxP2. Our finding that MOR1 immunolabeling intensity in the striatum of *Cnr1^−/−^* KO mice was not significantly different from controls (Fig. 6*A–D*) enabled us to use MOR1 as a marker to identify striosomal and matrix areas in all of these assays.

**Figure 7.**
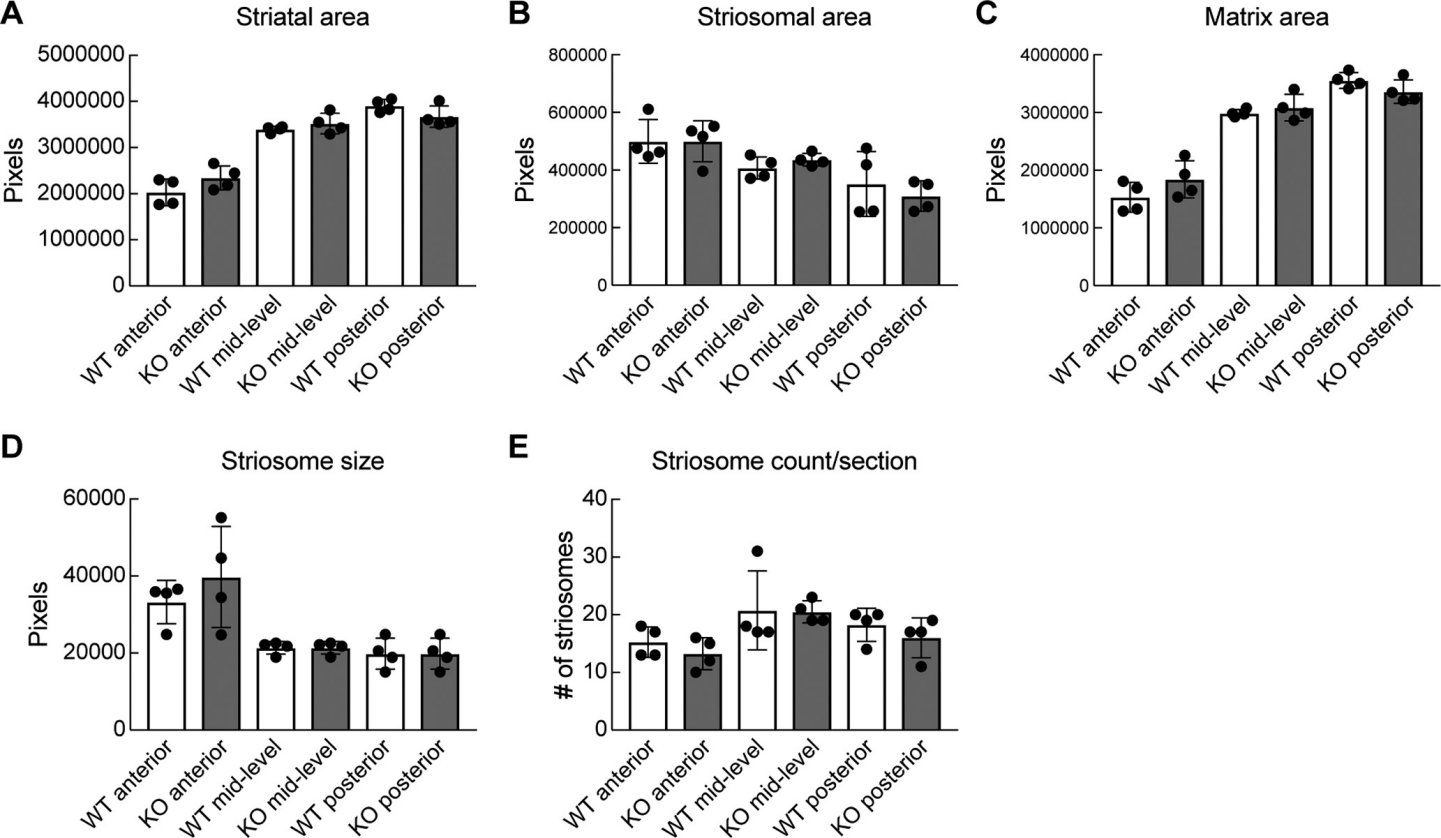
Striosome-matrix architecture in adult *Cnr1^−/−^* KO mice. MOR1 immunolabeling in coronal sections at matched levels was used to test for genotype differences in the total area of the striatum (***A***), striosomes (***B***), and matrix (***C***) and for measuring average striosome size (***D***) and density (***E***). No significant genotype differences were found (*p *>* *0.05 for all comparisons). Means for each animal and SDs of interanimal variability are plotted.

In *Cnr1^−/−^* KO mice, we observed that MOR1-positive clusters were perturbed at the dorsolateral aspect of the caudoputamen, in the region where abnormally bunched corticofugal fibers enter the striatum, as previously described for *Cnr1^−/−^* KO mice (Berghuis et al., 2007; Mulder et al., 2008; Wu et al., 2010; Alpár et al., 2014; Saez et al., 2020; Fig. 6*B*). However, we found no significant differences in the total striatal, striosomal and matrix areas and in the striosome size and counts per section between *Cnr1^−/−^* KO and control mice when matched levels were compared (*p *>* *0.05 for all genotype comparisons by Student’s *t* test, N = 4 mice per genotype, *n *=* *3 matched-level slices per mouse; Fig. 7). As expected, the average MOR1-labeled areas were larger on average in the anterior part than in the posterior part of the striatum, and this was true for both genotypes (Fig. 7*B*,*D*; Miyamoto et al., 2018).

**Figure 8.**
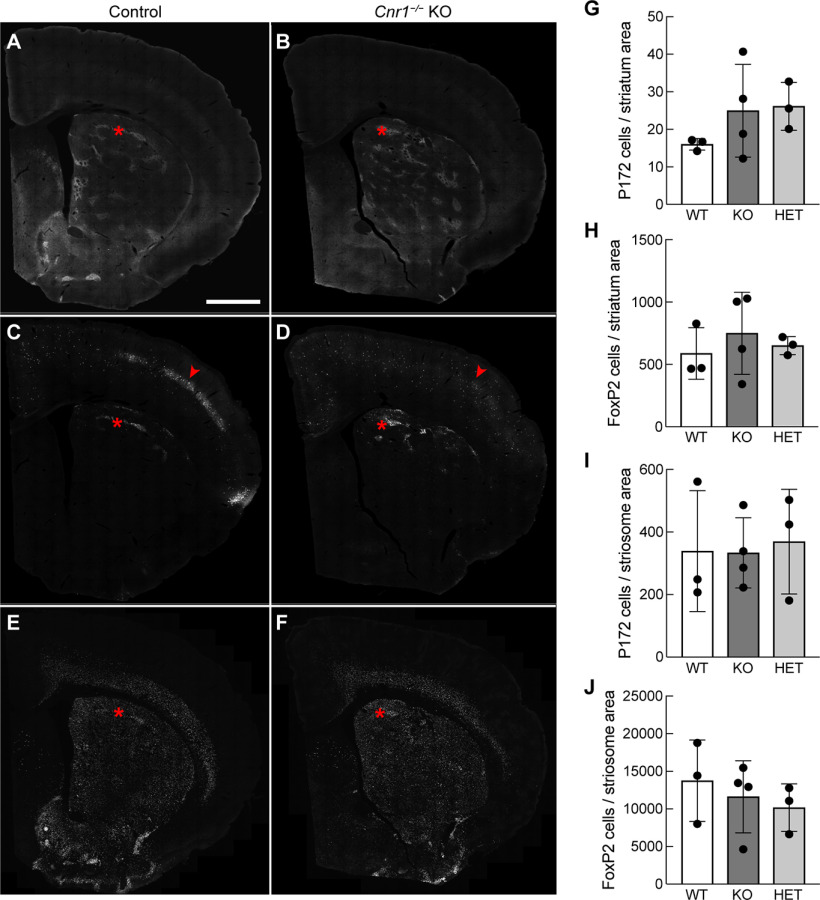
Striosomal cell density in adult *Cnr1^−/−^* KO mice. Samples of striatal sections triple-labeled for MOR1 (***A***, ***B***), P172-mCitrine (***C***, ***D***), and FoxP2 (***E***, ***F***) that were used for counting striosomal cell densities within striosomes and across the dorsal striatum. Red asterisks highlight single striosomes. Note the absence of P172-mCitrine-labeled cells in cortical layer 4 in *Cnr1^−/−^* KO mice [region designated by a red arrowhead in control (***C***) and KO (***D***)]. Scale bar: 500 μm (***A*** and applies to all panels). Graphs show the number of cells labeled for the striosomal cell markers P172-mCitrine (***G***, ***I***) and FoxP2 (***H***, ***J***), normalized to striatal (***G***, ***H***), or striosomal (***I***, ***J***) area (arbitrary units). Cells were counted for each of four level-matched sections from control (*N *=* *3), *Cnr1^−/−^* KO (*N *=* *4), and *Cnr1^−/+^* HET (*N *=* *3) mice. For all comparisons to controls, *p* > 0.05 by ANOVA with Dunnett’s correction. Means for each animal and SDs of interanimal variability are plotted.

As a further test of striatal compartmentalization, we counted the number of striosomal cell bodies that were positive for P172-mCitrine-immunolabeling or FoxP2-immunolabeling in sections from adult *Cnr1^−/−^* KO, *Cnr1^−/+^* heterozygous and sibling control mice. We found that the densities within MOR1-positive striosomes and across the whole striatum were not significantly different among genotypes (Fig. 8*A–J*). These counts of putative striosomal neurons still do not address subtle changes in the disposition of the striosomes as seen in cross-section or as seen in 3D movies (e.g., Movie 2) in *Cnr1^−/−^* KO mice, particularly at the dorsolateral border with the white matter, which might be related to the abnormal patterning of cerebral cortical and thalamic axons that course through the striatum. Our findings nevertheless indicate that CB1R signaling is required neither for the ultimate formation of striosomal neurons, nor for their organization into clusters (Matsushima and Graybiel, 2020). This situation stands in contrast to the well documented requirement for CB1R in cell neogenesis, survival or differentiation in other brain regions including the cerebral cortex (Gaffuri et al., 2012), a phenotype evidenced by the paucity of P172-mCitrine-positive cells that we observed in cortical layer 4 (Shima et al., 2016) of *Cnr1^−/−^* KO mice (Fig. 8*C*,*D*).

### In *Cnr1^−/−^* KO mice, striosomal and dopamine-containing neurons successfully reached their long-range targets via the striatonigral and nigrostriatal tracts

**Figure 9.**
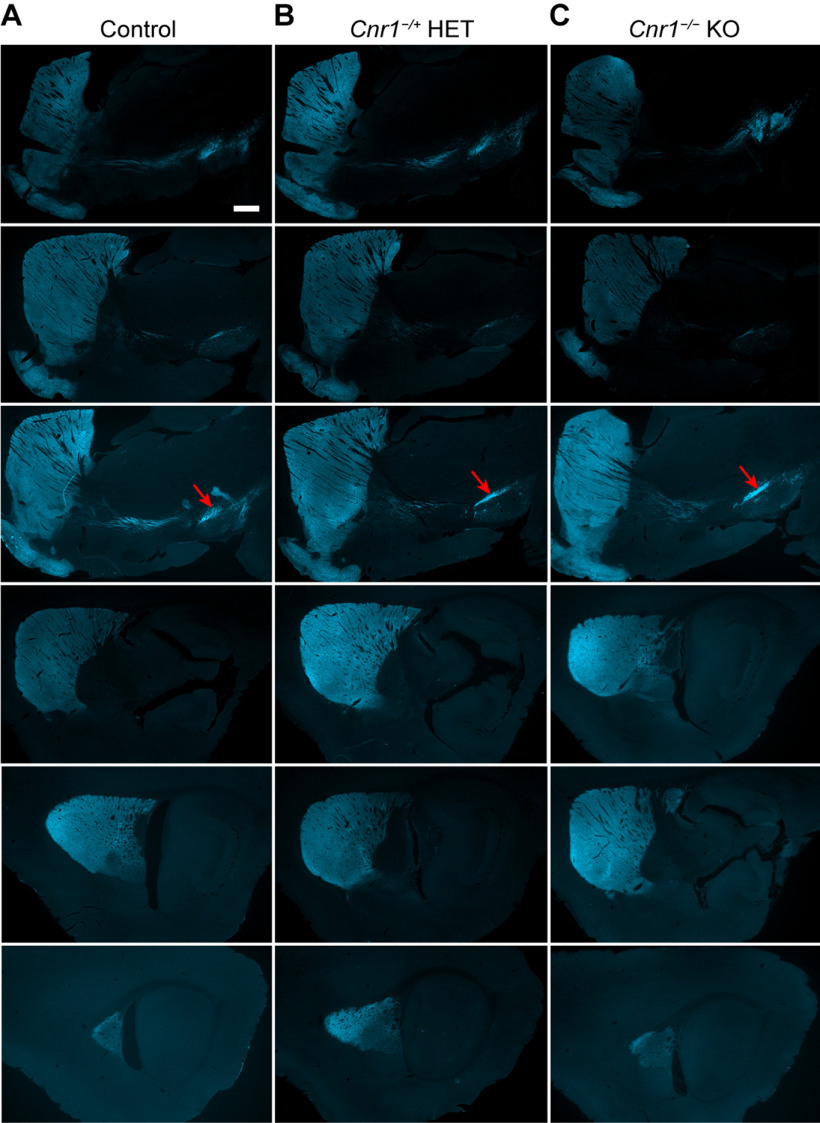
Nigrostriatal innervation in adult *Cnr1^−/−^* KO mice. Serial sagittal sections through the basal ganglia of control (***A***), *Cnr1^−/+^* heterozygous (***B***), and *Cnr1^−/−^* KO (***C***) mice immunolabeled for DAT (cyan) highlight the dopaminergic projections from the midbrain that innervate the entire striatum. Anterior is to the left, and sections run from medial (top) to lateral (bottom). The ventral tier dopaminergic neurons are indicated by a red arrow. Scale bar: 500 μm (upper left panel and applies to all panels). *N *=* *5 mice imaged for each genotype (one mouse for each genotype shown here).

**Figure 10.**
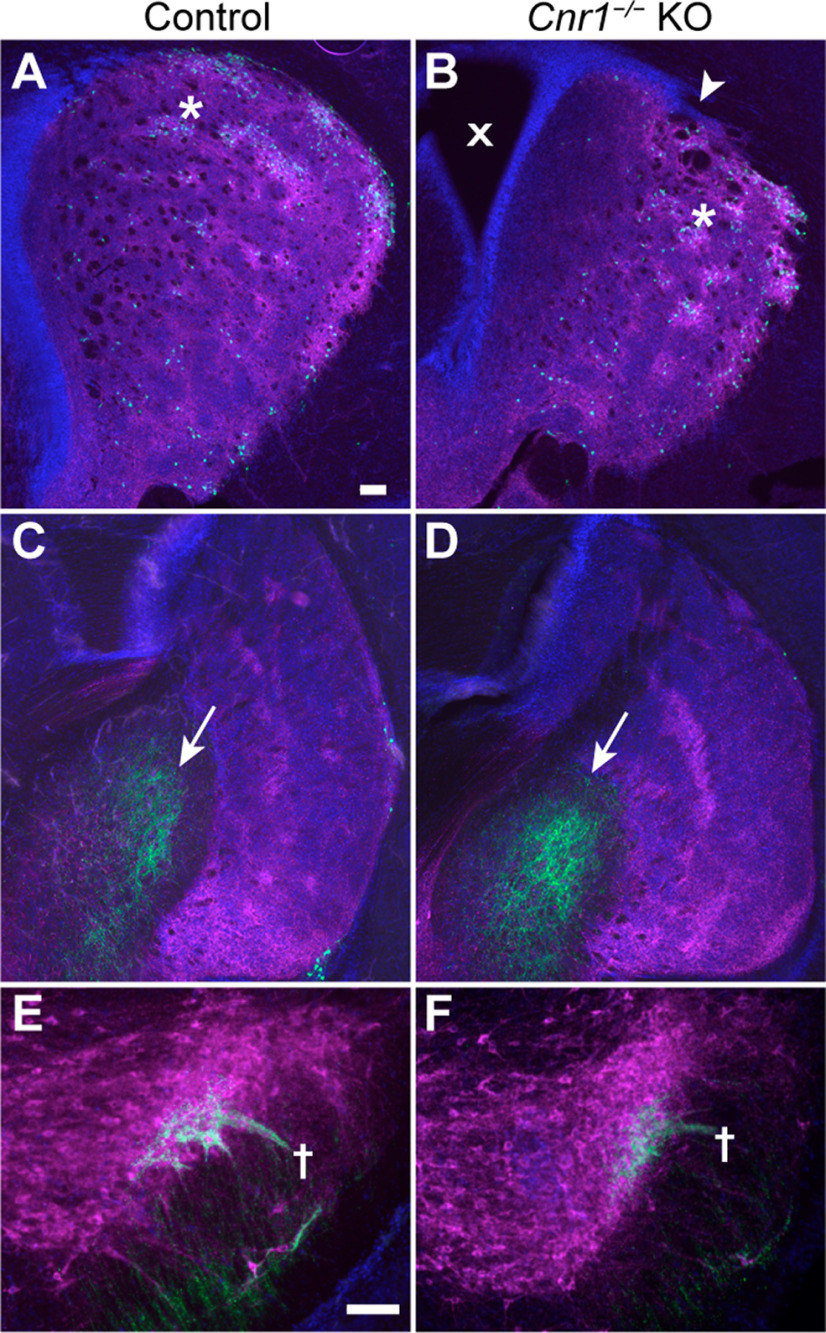
Developing striosomes and dopamine islands in *Cnr1^−/−^* KO mouse pups. Serial coronal sections through the basal ganglia of P5 controls and *Cnr1^−/−^* KO pups carrying the P172-mCitrine striosomal marker (green) are immunolabeled for the dopaminergic cell marker TH (magenta). Medial is to the left, lateral to the right for each panel. Dopamine islands (white asterisks in top panels) corresponding to developing striosomes are enriched for TH fibers and P172-mCitrine-labeled (mostly dorsal) striosomal neurons in the anterior (***A***, ***B***) and posterior (***C***, ***D***) striatum. A white arrowhead (***B***) indicates where the striatum is disrupted by bunched corticofugal fibers piercing the dorsolateral striatum, a known *Cnr1^−/−^* KO phenotype (Wu et al., 2010). The enlarged ventricles (marked by an x in ***B***) and laterally confined striosomes and dopamine islands suggest delayed striatal development in some *Cnr1^−/−^* KO pups relative to sibling controls, but this was not further investigated. Striosomal fibers reaching the external globus pallidus (marked by arrows in ***C***, ***D***) are evident in both genotypes by P172-mCitrine labeling (***C***, ***D***). ***E***, ***F***, The proximity of striosomal axons and dopaminergic fibers in bouquets is evident in controls (unresolved green and magenta labeling appears white), but in *Cnr1^−/−^* KO pups the co-labeled bundles are less distinct (developing bouquet stems are indicated by crosses). Scale bars: 100 μm. The scale bar in ***A*** applies to ***A***–***D***, and the scale bar in ***E*** applies to ***E*** and ***F***. *N *=* *4 pups examined for each genotype. Cell nuclei are labeled by DAPI (blue).

The dopamine-containing neurons of the ventral tier SNc, located among the poorly organized striosomal axon bundles in *Cnr1^−/−^* KO mice, are known to send long-range axonal projections to the dorsal striatum, with a preference for striosomes (Matsuda et al., 2009; Crittenden et al., 2016; Sgobio et al., 2017). A smaller proportion of dopamine-containing inputs to the dorsal striatum arise from the ventral tegmental area of the midbrain, which was not studied here. In adult *Cnr1^−/−^* KO mice, the nigrostriatal projections appeared generally normal, based on immunolabeling for DAT (Fig. 9*A–C*). In newborn mice, dopamine-containing inputs preferentially target developing striosomes to form tyrosine hydroxylase (TH)-positive “dopamine islands” (Graybiel, 1984; Miura et al., 2007). We did not perform quantitative analyses, but we did observe typical distributions of TH-positive dopamine islands that corresponded to striosomes based on overlapping distribution of P172-mCitrine-positive cells in P5 control and *Cnr1^−/−^* KO brains (Fig. 10*A–D*). We detected striosome-nigral development with P172-mCitrine and MOR1 immunomarkers for striosomal axons and TH for dopaminergic axons and dendrites; DAT expression was not used because it was not enriched in dopaminergic nigral processes of the early postnatal mice. It was clear that burgeoning bouquets were present in the SN with both striosomal axon and dopaminergic dendrite markers, in P5 *Cnr1^−/−^* KO pups (Fig. 10*E*,*F*).

### Striosome-dendron bouquets formed, and became enriched for CB1R, between P5 and P7

**Figure 11.**
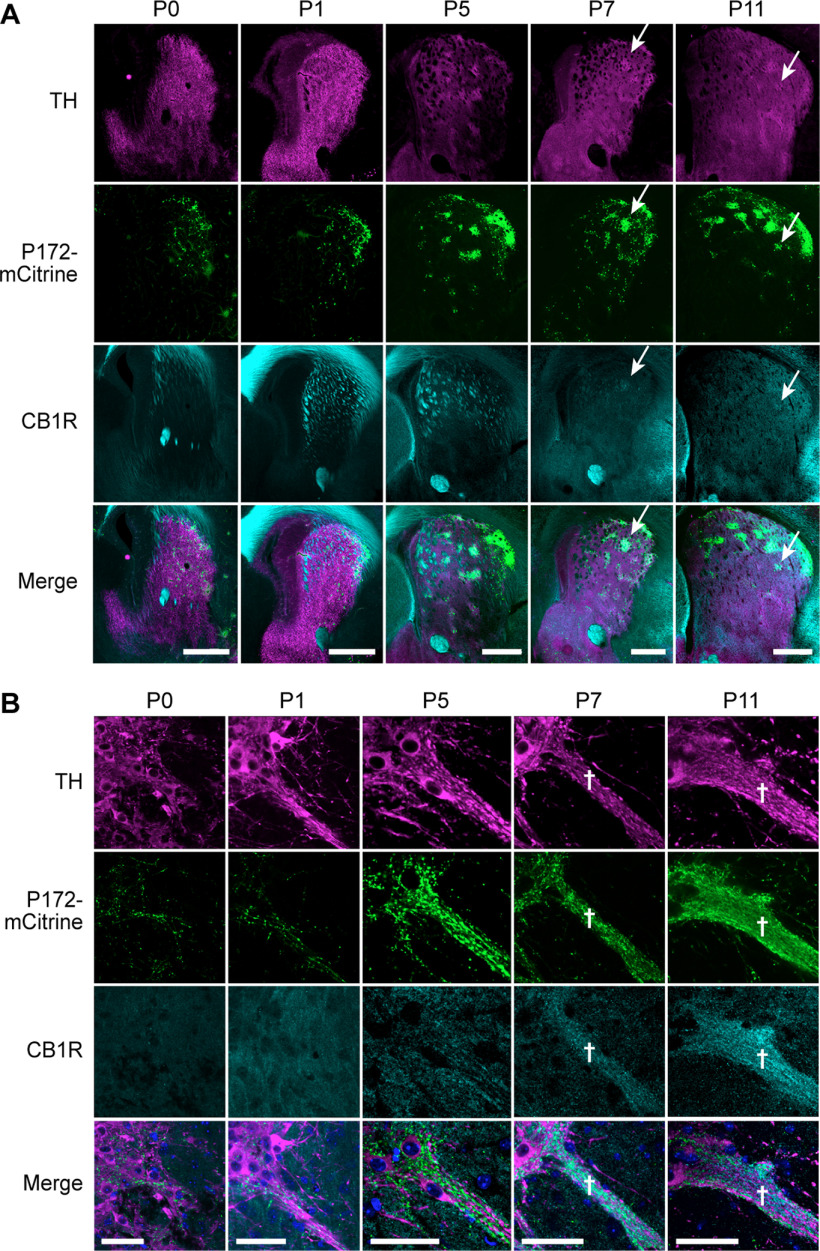
Bouquet formation in neonatal mice coincides with CB1R enrichment in striosomes. ***A***, Coronal sections through the left striatum in P0–P11 mouse pups, co-labeled to identify dopamine islands that innervate developing striosomes (TH) and striosomal markers (P172-mCitrine and CB1R). CB1R enrichment in striosomes is first apparent in the sections from P7 pups (white arrows in P7 and P11 panels), which corresponds to its enrichment in the expanding bouquet “stem” (designated by white crosses in panel ***B***). White arrows designate a single striosome labeled for TH, P172 and CB1R in P7 and P11 pup brains. Scale bars: 500 μm (shown in the merged images panel). *N* = 4 pups for each age. ***B***, Coronal sections through the left SN show single bouquets, from P0–P11 mouse pups, co-labeled to identify dopaminergic dendrites (TH), and striosomal axons (P172-mCitrine and CB1R). CB1R expression onset in striosomal fibers near the TH-rich SNcv border and along ventrally extending TH-positive fiber bundles appears to correlate with the enlargement of the bouquet “stem” (defined by white crosses in P7 and P11 panels). Scale bars: 50 μm (shown in the merged images panel). *N *=* *4 pups for each age.

To test for a potential influence of CB1R expression on the initial formation of bouquets, we determined when striosomes and bouquets were forming in developing control pups and when CB1R expression began in incoming striosomal axons destined to innervate the bouquet dendrons. In P0 to P11 pups, we found that we could track the initial formation and progressive formation of striosomes and growth of bouquet stems (Fig. 11*A*,*B*). Remarkably, expression of CB1R in striosomes and striosome-nigral axons (labeled for P172-mCitrine) was not yet visible at P5 (Fig. 11*A*,*B*), despite the abundance of CB1R in corticofugal fibers from P0 to P5 (Harkany et al., 2007). By P7, however, CB1R expression in corticofugal fibers passing through the striatum was diminished and CB1R expression in striatal neuropil, with enriched expression in striosomes, became apparent (Fig. 11*A*). Correspondingly, enrichment for CB1R within striosomal axons in bouquet stems and at the SNcv/SNr border, relative to the surrounding nigral neuropil, also became apparent by P7 (Fig. 11*B*).

**Figure 12.**
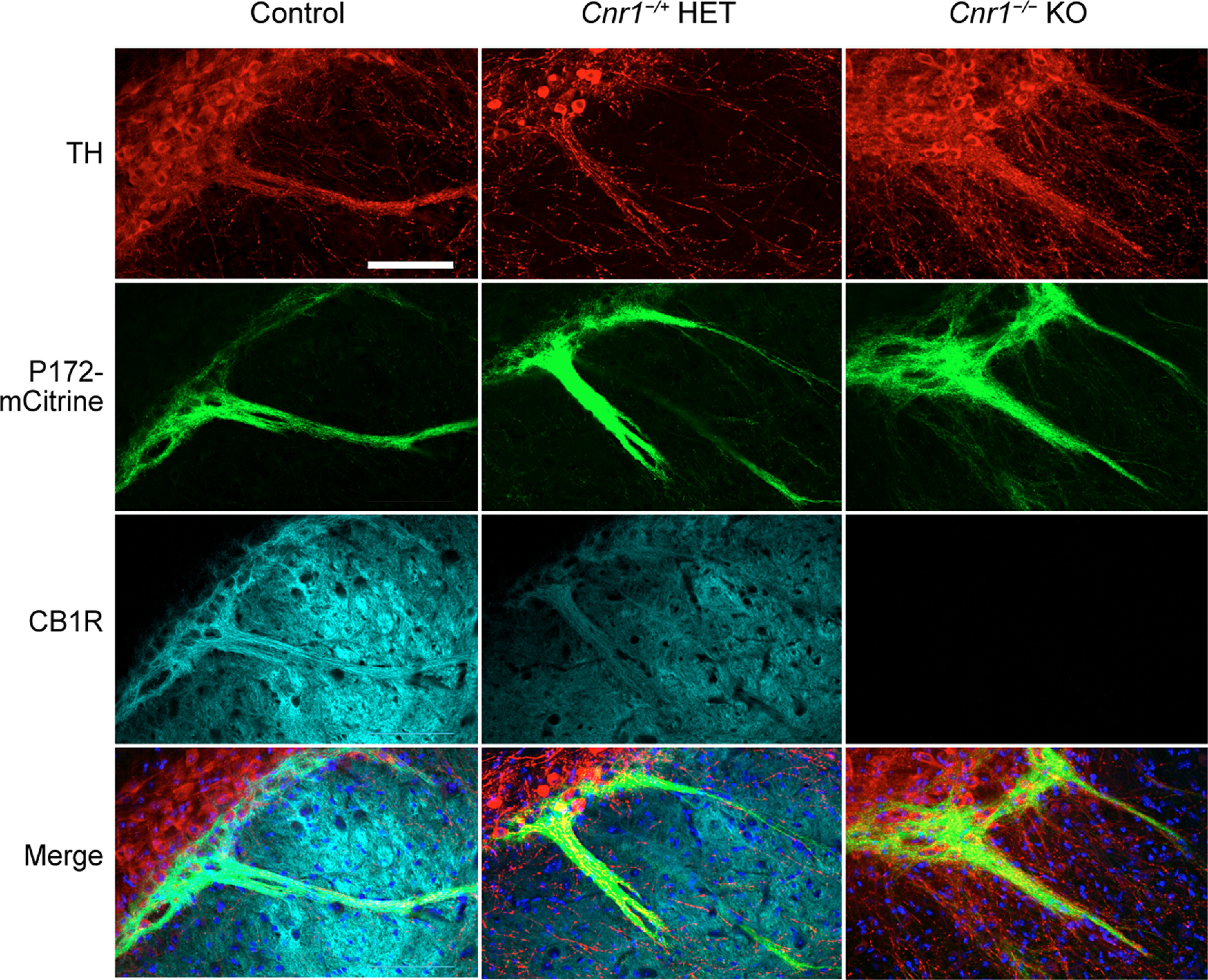
CB1R antibody specificity. The left SN of adult *Cnr1^+/+^* control, *Cnr1^−/+^* heterozygous (HET), and *Cnr1^−/−^* KO mice carrying the *P172-mCitrine* striosomal marker were immunolabeled for CB1R and the dopaminergic cell marker TH, confirming the immunospecificity of the CB1R antibody used in this study. *N* > 3 mice for control and KO genotypes. Scale bar: 100 μm (upper left panel and applies to all panels).

We considered that the gradual postnatal enrichment of CB1R in developing bouquets might simply be a function of striosomal axon density, rather than rising expression in each fiber. However, the expression of P172-mCitrine was strongly expressed in bouquets already by P5, and the gradual striosomal enrichment of CB1R expression at the level of the striatum during this early postnatal period (Fig. 11*A*) further indicated that it was not until after P5 that CB1R became enriched in striosomal neurons and their nigral projections. A key control supporting this conclusion was confirmation of the specificity of the CB1R antibody that we used. We found strong CB1R antibody immunoreactivity in adult controls, with slightly reduced immunoreactivity in *Cnr1^−/+^* heterozygous mice and no labeling in *Cnr1^−/−^* KO mice (Fig. 12).

**Figure 13.**
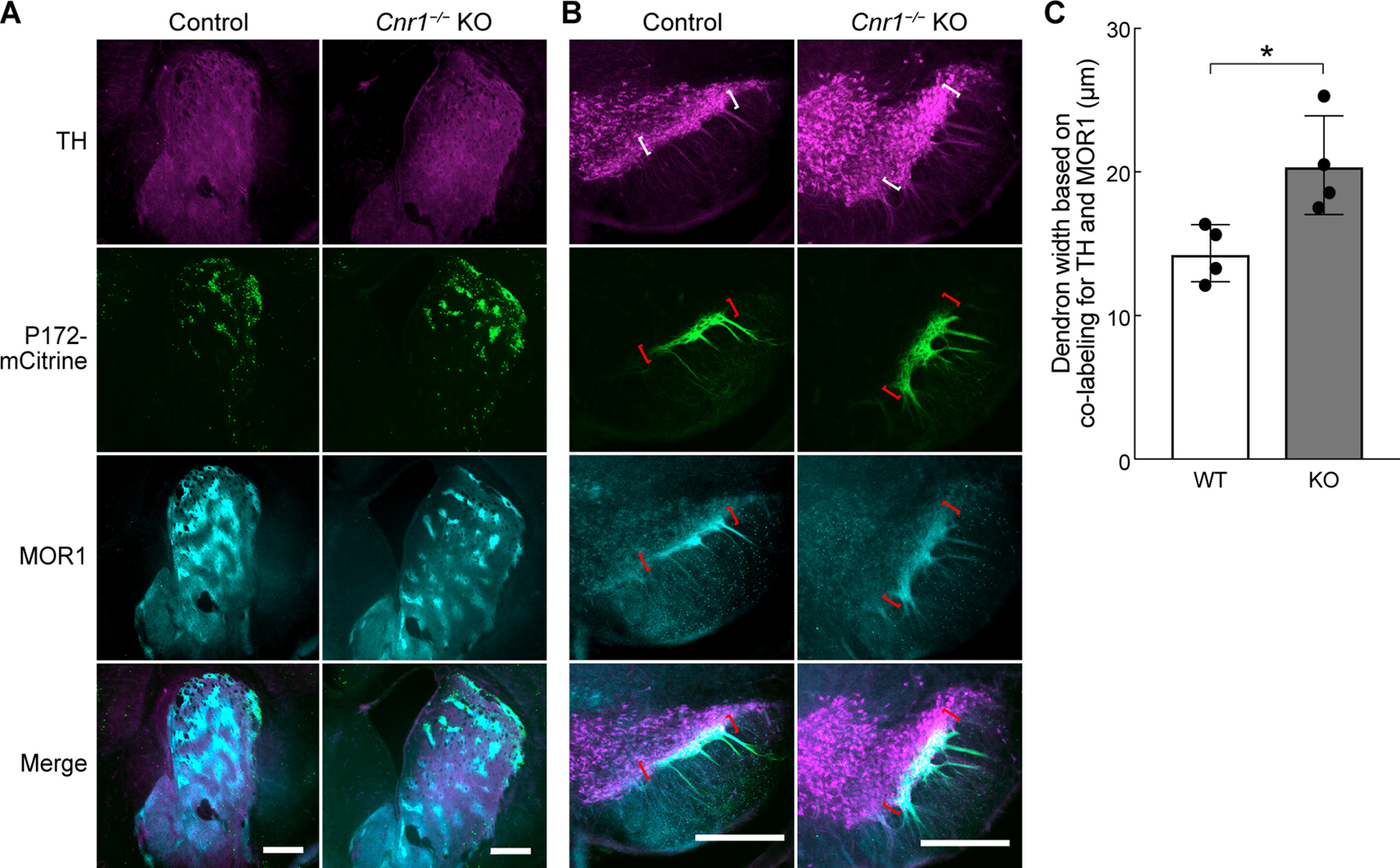
Developing bouquets are abnormal in *Cnr1^−/−^* KO developing mice. ***A***, ***B***, Coronal hemi-sections through the left striatum (***A***) and SN (***B***) of P11 pups carrying the P172-mCitrine striosomal marker (green) are immunolabeled for the dopaminergic cell marker TH (magenta) and striosomal projection neuron marker MOR1 (cyan). Similar to the adult, the striosome organization appears grossly normal in *Cnr1^−/−^* KO mice at this intermediate stage of bouquet development, but the striosomal and dopaminergic fibers appear bunched up rather than forming a discrete border (region in colored brackets) between the SNcv and the SNr as they do in control mice. *N *=* *4 mice for each genotype. Scale bars: 500 μm (shown in the merged images panel). ***C***, The average dorsoventral thickness of the SNcv border, defined as immunopositive for both MOR1 and TH, was significantly greater as measured in coronal nigral sections from *Cnr1^−/−^* KO pups compared with controls (*p = 0.029 by Student’s unpaired *t* test, *N *=* *4 mouse hemispheres per genotype). Means for each animal and SDs of interanimal variability are plotted. Images from which measurements were made are shown in Extended Data Figure 13-1*A*,*B*.

Finally, we tested whether the bouquet phenotype that we discovered in adult *Cnr1^−/−^* KO mice was already evident at ages corresponding to the onset of CB1R enrichment in striosomal axons. In P11 pups, the striosomal axons and dopamine-containing dendrites clearly appeared piled up at the SNcv/SNr border (Fig. 13*A*,*B*). We quantified this phenotype by measuring, genotype-blind, the dorsal-ventral distance of the region co-labeled for MOR1 and TH and found that there was a significantly thicker SNcv/SNr border region in P11 *Cnr1^−/−^* KO mice than in controls (*p *=* *0.029 by unpaired Student’s *t* test, *N *=* *4 mice per genotype, *n *=* *1 hemisphere slice per mouse pup; Fig. 13*C*). Together, these results suggest that CB1R expression begins in striosomal axons postnatally, during bouquet formation, and is required for striosomal axons and their terminals to communicate normally with dopamine-containing dendrites by forming discrete bouquets.

10.1523/ENEURO.0318-21.2022.f13-1 Extended Data Figure 13-1 Coronal sections through the left nigra of controls (***A***) and sibling *Cnr1^−/−^* KO (***B***) pups. Co-labeling for TH and MOR1 was used to measure the thickness of the SNcv, defined by double-labeled region and designated by yellow line. Scale bars: 50 μm (shown in the merged images panel). Download Figure 13-1, TIF file.

## Discussion

### CB1R in neurodevelopment of striosome-dendron bouquets

Our findings lead to two main conclusions. First, it is in the early postnatal period that the elaboration of striosome-dendron bouquets occurs in the substantia nigra. These consist of clusters of dopamine-containing nigral neurons, located within the SNcv along the border with the SNr, that intertwine their proximal and ventrally extending dendrites with one another and with incoming striatal afferent fibers from striosomes. This close intertwining of striosomal inputs allows powerful control of the ventral nigral tier by clusters of striosomal neurons in the striatum. Thus, this critical early postnatal period is one of growth, but also a time of vulnerability, of the striatonigral circuit. Our second conclusion, based on observations in *Cnr1^−/−^* KO mice lacking expression of CB1R, is that the endocannabinoid receptor CB1R exerts a critical and essential influence on this postnatal development of striosome-dendron bouquets. The incoming striosomal axons and the ventral dopamine-containing neurons fail to form fully organized bouquets, resulting in a pileup of incoming striosomal axons at the ventral tier border with the SNr. This finding serves as an alert that interfering with CB1R signaling, including here the *Cnr1* genetic deletion mimicking CB1R antagonism, could distort dopamine signaling beginning in the first days of postnatal development and into adulthood.

The ventral tier nigrostriatal dopamine-producing neurons are thought to modulate distinct aspects of motor control and behavior both by means of dopamine release in the dorsal striatum and by local dopamine release within the substantia nigra, including within the SNr that lies ventral to the SNcv and is penetrated by descending dopaminergic dendrites (Mukhida et al., 2001; Witkovsky et al., 2009; Zhou et al., 2009; Rice and Patel, 2015; Robinson et al., 2019; Salvatore et al., 2019; González-Rodríguez et al., 2021). Thus, disruption of striatonigral CB1R signaling in newborns could have profound long-term effects on dopamine-dependent behaviors; these could include not only movement-related activity but also aspects of mood, motivation and possibly habit formation including habitual drug abuse. These are important issues to address. Whether ligand-mediated overactivation or blockade of CB1R in the early postnatal period has neurodevelopmental consequences for the striatonigral system has not been addressed by our findings, but both conditions are of great public health consequence because cannabinoids, such as those in marijuana, are readily transmitted to nursing infants via mother’s milk and treatment with either agonists or antagonists of CB1R could possibly affect dopamine-dependent modulation of behavior and neural circuit function (Schneider, 2009; Scheyer et al., 2020).

Despite the deformations in the organization of striosomal axons and dopamine-containing dendrites within the substantia nigra of *Cnr1^−/−^* KO mice, we did not observe gross changes in the density of striosomal fibers within the striatonigral tract that extends to this midbrain target. Nor did we observe gross changes in the density of dopaminergic axons in the nigrostriatal tract reaching the striatum. These findings are consistent with there not being significant changes between *Cnr1^−/−^* KOs and controls in the number of striosomal neurons, based on our area measurements and cell body counts with the striosome markers MOR1, P172-mCitrine and FoxP2 or, in previous work, the number of TH-positive dopaminergic neurons in the substantia nigra (Steiner et al., 1999; Gargano et al., 2020). However, we did see deformation of the dorsolateral aspect of the caudoputamen, in regions where at minimum the striosomal so-called streak at the edge of the striatum could have been affected. Our finding that striosomal axons did reach the substantia nigra, although failing to form normal bouquets, strongly suggests that CB1R function is required for the normal juxtaposition of striosomal and dopaminergic fibers within the midbrain, but that it is not essential for their long-range pathfinding. This conclusion is striking, given evidence from study of the birthdate-dependent development of the striosomal innervation of the bouquets that the long-distance trajectory and final arborization patterns of the striosome-nigral innervations are under separate control (Matsushima and Graybiel, 2020).

### CB1R in prenatal and postnatal axon pathfinding

During development of the cerebral cortex and hippocampus in rodents, CB1R is less strongly expressed in the postnatal period than it is prenatally, when it has key functions in neuronal proliferation, migration and axon pathfinding for neurons and interneurons. Long-range guidance of cerebral corticofugal axons during embryonic development (Alpár et al., 2014; Saez et al., 2020) is thought to involve a meeting known as a “handshake” mechanism that occurs near the border of the striatum and the corpus callosum (Wu et al., 2010). When CB1R-expressing corticofugal axons extend ventrally into the striatum, they encounter endocannabinoid-releasing thalamocortical axons that are themselves extending dorsally into the striatum. CB1R-dependent endocannabinoid signaling between the two axonal types has been reported to allow the developing fiber connections to find their respective targets (Wu et al., 2010). In *Cnr1^−/−^* KO adult and P5 mice, the cortical fibers fail to find their targets and appear bunched up near the border between striatum and corpus callosum as previously shown and as seen in Figures 6*B*, 10*B*.

Whether the dendrites of the dopamine-containing neurons of the developing bouquet stems produce or release endocannabinoids is unknown. However, at maturity, dopamine-containing neurons in the nearby ventral tegmental area express endocannabinoids that signal to CB1R on input axons from the nucleus accumbens septi (Riegel and Lupica, 2004). Based on this biological principal, we hypothesize that, during development, CB1R-positive striosomal axons “handshake” with endocannabinoid-producing dendrites from dopaminergic SNcv neurons to guide the tight intertwining of striosomal axons and dopaminergic dendrites that form the normally discrete border between the SNcv and SNr and the ventrally extending bouquet dendrons. Our data in mice suggest that this signaling handshake occurs postnatally, as CB1R expression becomes enriched in striosomes and their striatonigral axons between P5 and P7, and loss of CB1R gives rise to measurably abnormal bouquets by P11. Our measurements indicated that in *Cnr1^−/−^* KO adult and P11 mice, the intertwined striosomal and dopaminergic fibers formed a thicker SNcv tier, and they appeared less discrete and more loosely bundled along the border with the SNr. Expression of the striosomal axon marker MOR1 was significantly lower in bouquet stem dendrons in the left hemisphere, with a similar trend for the right, in adult *Cnr1^−/−^* KOs relative to controls, consistent with the observed sparsity of dendrons in some *Cnr1^−/−^* KO mice. Together, these results suggest that, in the absence of CB1R, striosomal axons reach the midbrain but become bunched along the SNcv/SNr border rather than organizing with dopaminergic fibers to form discrete bouquets and ventrally extending dendrons.

By tracing of single fibers in aged *P172-mCitrine* mice, we observed one that appeared to climb dorsally along the stem and to form terminal branches and bulbs along the SNcv/SNr border. Whether all striosomal fibers follow the same path to form bouquets remains unknown. In the absence of CB1R, signaling between striosomal axons and dopamine-containing dendrites could fail, and normal connections could be disrupted as a consequence, potentially contributing to the disorganized SNcv in *Cnr1^−/−^* KO mice. We noted that it was along architectural borders, both for the striatum and the nigra, that the loss of CB1R expression had the most obvious impact.

An alternative accounting for the abnormal striosome-dendron bouquet phenotype in the *Cnr1^−/−^* KO mice is that there is a temporal mismatch of the connections. For example, if striatal projection neurons undergo delayed maturation, they might not reach their normal nigral destination during the appropriate time-window to form connections with the dopamine-containing dendrites that are developing. Other caveats include whether the phenotype of abnormal striosome-dendron bouquet formation results from the loss of CB1R expression in striosomes, or whether it is an indirect outcome from CB1R loss in another cell type. It is possible that the phenotype is an effect of another perturbation that affects both the afferents and the dendrites. We have not yet been able to determine answers to these crucial issues; further research focused on this neurodevelopmental phenotype could be mapped to a given cell type. Regardless of how CB1R regulates cellular functions and in which cell types, however, the finding that CB1R plays a key role in neurodevelopment of the striatonigral system will need to be taken into account when evaluating phenotypes in mature *Cnr1^−/−^* KO mice and clinically related findings.

### CB1R in striosome-dendron bouquets of adults

Disruption of CB1R function specifically in adults could as well influence striosomal control of nigral dopamine cells, as CB1R is strongly enriched in striosome-dendron bouquets of mature mice (Davis et al., 2018). CB1R signaling in adult mice is reported to control neuronal plasticity in the striatum, midbrain, hippocampus, cerebral cortex, amygdala and cerebellum (Augustin and Lovinger, 2018; Busquets-Garcia et al., 2018). Agonism of presynaptic CB1R can inhibit release of glutamate and GABA from axonal terminals of numerous cell types, including axon terminals from the ventral striatum that disinhibit dopaminergic neurons in the midbrain ventral tegmental area (Riegel and Lupica, 2004; Domenici et al., 2006; Oleson et al., 2014; Tung et al., 2016; Covey et al., 2017; Augustin and Lovinger, 2018). In contrast to striosomal neurons, dopamine-containing neurons express minimal CB1R (Julian et al., 2003). Therefore, CB1R agonism might regulate dopaminergic cell activity via blocking the inhibition and rebound activation from striosomal axons, which are normally mediated by their release of GABA (Lee and Tepper, 2009; McGregor et al., 2019; Evans et al., 2020). Notable complexities to these proposed effects include that striosomal axons co-express the excitatory slow-acting peptide substance P (Bolam and Smith, 1990; Steiner et al., 1999). Further, CB1R is known to regulate peptide release from non-neuronal cell types (Malenczyk et al., 2013). Whereas activation of CB1R on axon terminals inhibits neurotransmitter release, CB1R activation of astrocytes potentiates glutamate release from surrounding neurons (Navarrete and Araque, 2010). Thus, CB1R can both activate and inhibit neurotransmitter release, depending on its site of action (Stella, 2010). Considering that astrocytes are embedded within bouquet stems (Crittenden et al., 2016), CB1R might regulate bouquet functions by potentiating neurotransmitter release as well as by disinhibition.

Finally, it is notable that striatal expression of CB1R is dysregulated in both Parkinson’s disease (Zeng et al., 1999; Hurley et al., 2003; Navarrete et al., 2018) and Huntington’s disease (Glass et al., 2000; Van Laere et al., 2010), adult-onset disorders with striosomal abnormalities (Crittenden and Graybiel, 2011), dopamine dysregulation and complex motor and mood dysfunction. As a consequence, CB1R has been targeted for preclinical intervention (Kreitzer and Malenka, 2007; Blázquez, et al., 2011; Naydenov et al., 2014). The developmental effects that we report here could yield important clues for work on these and other conditions in which CB1Rs, alone or in conjunction with others, influence transmission in the nigrostriatal dopamine system.

## Synthesis

Reviewing Editor: Kristen Harris, The University of Texas at Austin Decisions are customarily a result of the Reviewing Editor and the peer reviewers coming together and discussing their recommendations until a consensus is reached. When revisions are invited, a fact-based synthesis statement explaining their decision and outlining what is needed to prepare a revision will be listed below. The following reviewer(s) agreed to reveal their identity: Rosario Moratalla.

General positive comments: The authors have been very responsive to the previous reviewers’ comments, resulting in a markedly improved manuscript. The report is now undergirded by clear rationale for the studies conducted, with better quantitative analysis and explanations of the results throughout. The data show an important role for CB1 receptors (CB1Rs), and therefore endogenous cannabinoids, in the development of striatonigral connections called striosome-dendron bouquets. Indeed, now-convincing anatomical data demonstrate that these structures are disrupted in mice lacking CB1Rs. Given that substantia nigra dopamine neuron activity is strongly regulated by striosome-dendron bouquets, the findings are important and should advance the field. The manuscript provides solid evidence for the implication of CB1R, first in the development of dopamine neurons of the midbrain and second in the connectivity between striatonigral GABAergic neurons within the striosomes with dopamine neurons in the SN. The authors now provide beautiful and convincing evidence that CB1 receptors (CB1Rs) are required for normal postnatal development of midbrain dopamine-containing neurons and their striatal inputs. Their findings not only shed light on the development of these cells and associated circuits, but also provide a warning for the use of therapeutic or recreational drugs that act at CB1Rs. Authors have done an excellent job in replying to the reviewers and have included additional data to further support their conclusions. Lovely images and excellent work.

Remaining items to resolve:

The 2 movies provided would be nice to have embedded in the paper

Page 2, line 19, striatonigral dopamine system. is it correct? Please check.

Fig 5, 8: Please add the asterisk in the figure to illustrate the statistical significance.

li. 25. “Surprising” would be better than “surprise” here.

Fig. 5A and 13C. Using “Diameter", “Distance", or “Thickness” (as in the text) might be clearer than “Depth” in the y-axis titles.

li. 247. P70 mice are adult, but not really “aged”. Better would be young adult, or similar, to avoid possible confusion with studies that examine the aging brain (e.g., in 1-2 year old mice).

li. 336-337. Referring to dopamine-containing neurons of the ventral tier of the SNc in the “the poorly organized striosomal neuropil in Cnr1−/− 337 KO mice” is confusing, as “striosomal neuropil” implies a striatal location. Also, the section immediately before this indicates that the organization of striatal striosomes is relatively normal.

## References

[B1] Alpár A, Tortoriello G, Calvigioni D, Niphakis MJ, Milenkovic I, Bakker J, Cameron GA, Hanics J, Morris CV, Fuzik J, Kovacs GG, Cravatt BF, Parnavelas JG, Andrews WD, Hurd YL, Keimpema E, Harkany T (2014) Endocannabinoids modulate cortical development by configuring Slit2/Robo1 signalling. Nat Commun 5:4421. 10.1038/ncomms5421 25030704 PMC4110686

[B2] Ashton JC, Wright JL, McPartland JM, Tyndall JD (2008) Cannabinoid CB1 and CB2 receptor ligand specificity and the development of CB2-selective agonists. Curr Med Chem 15:1428–1443. 10.2174/092986708784567716 18537620

[B3] Augustin SM, Lovinger DM (2018) Functional relevance of endocannabinoid-dependent synaptic plasticity in the central nervous system. ACS Chem Neurosci 9:2146–2161. 10.1021/acschemneuro.7b00508 29400439 PMC6720113

[B4] Berghuis P, Rajnicek AM, Morozov YM, Ross RA, Mulder J, Urbán GM, Monory K, Marsicano G, Matteoli M, Canty A, Irving AJ, Katona I, Yanagawa Y, Rakic P, Lutz B, Mackie K, Harkany T (2007) Hardwiring the brain: endocannabinoids shape neuronal connectivity. Science 316:1212–1216. 10.1126/science.1137406 17525344

[B5] Blázquez C, Chiarlone A, Sagredo O, Aguado T, Pazos MR, Resel E, Palazuelos J, Julien B, Salazar M, Börner C, Benito C, Carrasco C, Diez-Zaera M, Paoletti P, Díaz-Hernández M, Ruiz C, Sendtner M, Lucas JJ, de Yébenes JG, Marsicano G, et al. (2011) Loss of striatal type 1 cannabinoid receptors is a key pathogenic factor in Huntington’s disease. Brain 134:119–136. 10.1093/brain/awq278 20929960

[B6] Bolam JP, Smith Y (1990) The GABA and substance P input to dopaminergic neurones in the substantia nigra of the rat. Brain Res 529:57–78. 10.1016/0006-8993(90)90811-O 1704287

[B7] Busquets-Garcia A, Bains J, Marsicano G (2018) CB1 receptor signaling in the brain: extracting specificity from ubiquity. Neuropsychopharmacology 43:4–20. 10.1038/npp.2017.206 28862250 PMC5719111

[B8] Canales J, Graybiel AM (2000) A measure of striatal function predicts motor stereotypy. Nat Neurosci 3:377–383. 10.1038/73949 10725928

[B9] Covey DP, Mateo Y, Sulzer D, Cheer JF, Lovinger DM (2017) Endocannabinoid modulation of dopamine neurotransmission. Neuropharmacology 124:52–61. 10.1016/j.neuropharm.2017.04.033 28450060 PMC5608040

[B10] Crittenden JR, Graybiel AM (2011) Basal ganglia disorders associated with imbalances in the striatal striosome and matrix compartments. Front Neuroanat 5:59. 10.3389/fnana.2011.00059 21941467 PMC3171104

[B11] Crittenden JR, Tillberg PW, Riad MH, Shima Y, Gerfen CR, Curry J, Housman DE, Nelson SB, Boyden ES, Graybiel AM (2016) Striosome-dendron bouquets highlight a unique striatonigral circuit targeting dopamine-containing neurons. Proc Natl Acad Sci U S A 113:11318–11323. 10.1073/pnas.1613337113 27647894 PMC5056098

[B12] Crittenden JR, Lacey CJ, Weng FJ, Garrison CE, Gibson DJ, Lin Y, Graybiel AM (2017) Striatal cholinergic interneurons modulate spike-timing in striosomes and matrix by an amphetamine-sensitive mechanism. Front Neuroanat 11:20. 10.3389/fnana.2017.00020 28377698 PMC5359318

[B13] Crittenden JR, Gipson TA, Smith AC, Bowden HA, Yildirim F, Fischer KB, Yim M, Housman DE, Graybiel AM (2021a) Striatal transcriptome changes linked to drug-induced repetitive behaviors. Eur J Neurosci 53:2450–2468. 10.1111/ejn.15116 33759265 PMC8330602

[B14] Crittenden JR, Yoshida T, Davis MI, Graybiel AM (2021b) Immunofluorescence for free-floating brain sections. Protocols. Available at 10.17504/protocols.io.kracv2e.

[B15] Davis MI, Crittenden JR, Feng AY, Kupferschmidt DA, Naydenov A, Stella N, Graybiel AM, Lovinger DM (2018) The cannabinoid-1 receptor is abundantly expressed in striatal striosomes and striosome-dendron bouquets of the substantia nigra. PLoS One 13:e0191436. 10.1371/journal.pone.0191436 29466446 PMC5821318

[B16] Domenici MR, Azad SC, Marsicano G, Schierloh A, Wotjak CT, Dodt HU, Zieglgänsberger W, Lutz B, Rammes G (2006) Cannabinoid receptor type 1 located on presynaptic terminals of principal neurons in the forebrain controls glutamatergic synaptic transmission. J Neurosci 26:5794–5799. 10.1523/JNEUROSCI.0372-06.2006 16723537 PMC6675276

[B17] Evans RC, Twedell EL, Zhu M, Ascencio J, Zhang R, Khaliq ZM (2020) Functional dissection of basal ganglia inhibitory inputs onto substantia nigra dopaminergic neurons. Cell Rep 32:108156. 10.1016/j.celrep.2020.108156 32937133 PMC9887718

[B18] Everitt BJ (2014) Neural and psychological mechanisms underlying compulsive drug seeking habits and drug memories–indications for novel treatments of addiction. Eur J Neurosci 40:2163–2182. 10.1111/ejn.12644 24935353 PMC4145664

[B19] Gaffuri AL, Ladarre D, Lenkei Z (2012) Type-1 cannabinoid receptor signaling in neuronal development. Pharmacology 90:19–39. 10.1159/000339075 22776780

[B20] Gargano A, Beins E, Zimmer A, Bilkei-Gorzo A (2020) Lack of cannabinoid receptor type-1 leads to enhanced age-related neuronal loss in the locus coeruleus. Int J Mol Sci 22:5. 10.3390/ijms22010005 PMC7792602 33374940

[B21] Glass M, Dragunow M, Faull RLM (2000) The pattern of neurodegeneration in Huntington’s disease: a comparative study of cannabinoid, dopamine, adenosine and GABAA receptor alterations in the human basal ganglia in Huntington’s disease. Neuroscience 97:505–519. 10.1016/S0306-4522(00)00008-7 10828533

[B22] González-Rodríguez P, Zampese E, Stout KA, Guzman JN, Ilijic E, Yang B, Tkatch T, Stavarache MA, Wokosin DL, Gao L, Kaplitt MG, López-Barneo J, Schumacker PT, Surmeier JD (2021) Disruption of mitochondrial complex I induces progressive parkinsonism. Nature 599:650–656. 10.1038/s41586-021-04059-0 34732887 PMC9189968

[B23] Graybiel AM (1984) Correspondence between the dopamine islands and striosomes of the mammalian striatum. Neuroscience 13:1157–1187. 10.1016/0306-4522(84)90293-8 6152035

[B150] Graybiel AM, Moratalla R, Robertson HA (1990) Amphetamine and cocaine induce drug-specific activation of the c-fos gene in striosome-matrix compartments and limbic subdivisions of the striatum. Proc Natl Acad Sci U S A 87:6912–6916 2118661 10.1073/pnas.87.17.6912 PMC54648

[B24] Graybiel AM (2008) Habits, rituals, and the evaluative brain. Annu Rev Neurosci 31:359–387. 10.1146/annurev.neuro.29.051605.112851 18558860

[B25] Gremel CM, Lovinger DM (2017) Associative and sensorimotor cortico-basal ganglia circuit roles in effects of abused drugs. Genes Brain Behav 16:71–85. 10.1111/gbb.12309 27457495 PMC5503114

[B26] Harkany T, Guzmán M, Galve-Roperh I, Berghuis P, Devi LA, Mackie K (2007) The emerging functions of endocannabinoid signaling during CNS development. Trends Pharmacol Sci 28:83–92. 10.1016/j.tips.2006.12.004 17222464

[B27] Hurley MJ, Mash DC, Jenner P (2003) Expression of cannabinoid CB1 receptor mRNA in basal ganglia of normal and parkinsonian human brain. J Neural Transm (Vienna) 110:1279–1288. 10.1007/s00702-003-0033-7 14628192

[B28] Jedynak JP, Cameron CM, Robinson TE (2012) Repeated methamphetamine administration differentially alters fos expression in caudate-putamen patch and matrix compartments and nucleus accumbens. PLoS One 7:e34227. 10.1371/journal.pone.0034227 22514626 PMC3326007

[B29] Johansson PA, Andersson M, Andersson KE, Cenci MA (2001) Alterations in cortical and basal ganglia levels of opioid receptor binding in a rat model of l-DOPA-induced dyskinesia. Neurobiol Dis 8:220–239. 10.1006/nbdi.2000.0372 11300719

[B30] Julian MD, Martin AB, Cuellar B, Rodriguez De Fonseca F, Navarro M, Moratalla R, Garcia-Segura LM (2003) Neuroanatomical relationship between type 1 cannabinoid receptors and dopaminergic systems in the rat basal ganglia. Neuroscience 119:309–318. 10.1016/S0306-4522(03)00070-8 12763090

[B31] Koizumi H, Morigaki R, Okita S, Nagahiro S, Kaji R, Nakagawa M, Goto S (2013) Response of striosomal opioid signaling to dopamine depletion in 6-hydroxydopamine-lesioned rat model of Parkinson’s disease: a potential compensatory role. Front Cell Neurosci 7:74. 10.3389/fncel.2013.00074 23730270 PMC3656348

[B32] Kreitzer AC, Malenka RC (2007) Endocannabinoid-mediated rescue of striatal LTD and motor deficits in Parkinson’s disease models. Nature 445:643–647. 10.1038/nature05506 17287809

[B33] Lee CR, Tepper JM (2009) Basal ganglia control of substantia nigra dopaminergic neurons. J Neural Transm Suppl 73:71–90. 10.1007/978-3-211-92660-4_6 20411769

[B34] Liu QR, Canseco-Alba A, Zhang HY, Tagliaferro P, Chung M, Dennis E, Sanabria B, Schanz N, Escosteguy-Neto JC, Ishiguro H, Lin Z, Sgro S, Leonard CM, Santos-Junior JG, Gardner EL, Egan JM, Lee JW, Xi Z X, Onaivi ES (2017) Cannabinoid type 2 receptors in dopamine neurons inhibits psychomotor behaviors, alters anxiety, depression and alcohol preference. Sci Rep 7:17410. 29234141 10.1038/s41598-017-17796-y PMC5727179

[B35] Malenczyk K, Jazurek M, Keimpema E, Silvestri C, Janikiewicz J, Mackie K, Di Marzo V, Redowicz MJ, Harkany T, Dobrzyn A (2013) CB1 cannabinoid receptors couple to focal adhesion kinase to control insulin release. J Biol Chem 288:32685–32699. 10.1074/jbc.M113.478354 24089517 PMC3820903

[B36] Matsuda W, Furuta T, Nakamura KC, Hioki H, Fujiyama F, Arai R, Kaneko T (2009) Single nigrostriatal dopaminergic neurons form widely spread and highly dense axonal arborizations in the neostriatum. J Neurosci 29:444–453. 10.1523/JNEUROSCI.4029-08.2009 19144844 PMC6664950

[B37] Matsushima A, Graybiel AM (2020) Combinatorial developmental controls on striatonigral circuits. Cell Rep 31:107778. 10.1016/j.celrep.2020.107778 32553154 PMC7433760

[B38] McGregor MM, McKinsey GL, Girasole AE, Bair-Marshall CJ, Rubenstein JLR, Nelson AB (2019) Functionally distinct connectivity of developmentally targeted striosome neurons. Cell Rep 29:1419–1428.e5. 10.1016/j.celrep.2019.09.076 31693884 PMC6866662

[B39] Miura M, Saino-Saito S, Masuda M, Kobayashi K, Aosaki T (2007) Compartment-specific modulation of GABAergic synaptic transmission by mu-opioid receptor in the mouse striatum with green fluorescent protein-expressing dopamine islands. J Neurosci 27:9721–9728. 10.1523/JNEUROSCI.2993-07.2007 17804632 PMC6672981

[B40] Miyamoto S, Katayama S, Shigematsu N, Nishi A, Fukuda T (2018) Striosome-based map of the mouse striatum that is conformable to both cortical afferent topography and uneven distributions of dopamine D1 and D2 receptor-expressing cells. Brain Struct Funct 223:4275–4291. 10.1007/s00429-018-1749-3 30203304 PMC6267261

[B41] Moratalla R, Elibol B, Vallejo M, Graybiel AM (1996) Network-level changes in expression of inducible Fos-Jun proteins in the striatum during chronic cocaine treatment and withdrawal. Neuron 17:147–156. 10.1016/S0896-6273(00)80288-3 8755486

[B42] Mukhida K, Baker KA, Sadi D, Mendez I (2001) Enhancement of sensorimotor behavioral recovery in hemiparkinsonian rats with intrastriatal, intranigral, and intrasubthalamic nucleus dopaminergic transplants. J Neurosci 15:3521–3530. 10.1523/JNEUROSCI.21-10-03521.2001 PMC6762505 11331381

[B43] Mulder J, Aguado T, Keimpema E, Barabás K, Ballester Rosado CJ, Nguyen L, Monory K, Marsicano G, Di Marzo V, Hurd YL, Guillemot F, Mackie K, Lutz B, Guzman M, Lu HC, Galve-Roperh I, Harkany T (2008) Endocannabinoid signaling controls pyramidal cell specification and long-range axon patterning. Proc Natl Acad Sci U S A 105:8760–8765. 10.1073/pnas.0803545105 18562289 PMC2438381

[B44] Navarrete M, Araque A (2010) Endocannabinoids potentiate synaptic transmission through stimulation of astrocytes. Neuron 68:113–126. 10.1016/j.neuron.2010.08.043 20920795

[B45] Navarrete F, García-Gutiérrez MS, Aracil-Fernández A, Lanciego JL, Manzanares J (2018) Cannabinoid CB1 and CB2 receptors, and monoacylglycerol lipase gene expression alterations in the basal ganglia of patients with Parkinson’s disease. Neurotherapeutics 15:459–469. 10.1007/s13311-018-0603-x 29352424 PMC5935636

[B46] Naydenov AV, Sepers MD, Swinney K, Raymond LA, Palmiter RD, Stella N (2014) Genetic rescue of CB1 receptors on medium spiny neurons prevents loss of excitatory striatal synapses but not motor impairment in HD mice. Neurobiol Dis 71:140–150. 10.1016/j.nbd.2014.08.009 25134728 PMC4180675

[B47] Nelson A, Killcross S (2006) Amphetamine exposure enhances habit formation. J Neurosci 26:3805–3812. 10.1523/JNEUROSCI.4305-05.2006 16597734 PMC6674135

[B48] Oleson EB, Cachope R, Fitoussi A, Tsutsui K, Wu S, Gallegos JA, Cheer JF (2014) Cannabinoid receptor activation shifts temporally engendered patterns of dopamine release. Neuropsychopharmacology 39:1441–1452. 10.1038/npp.2013.340 24345819 PMC3988547

[B49] Pan C, Cai R, Quacquarelli FP, Ghasemigharagoz A, Lourbopoulos A, Matryba P, Plesnila N, Dichgans M, Hellal F, Ertürk A (2016) Shrinkage-mediated imaging of entire organs and organisms using uDISCO. Nat Methods 13:859–867. 10.1038/nmeth.3964 27548807

[B50] Prager EM, Plotkin JL (2019) Compartmental function and modulation of the striatum. J Neurosci 97:1503–1514. 10.1002/jnr.24522 31489687 PMC6801090

[B51] Rice ME, Patel JC (2015) Somatodendritic dopamine release: recent mechanistic insights. Philos Trans R Soc Lond B Biol Sci 370:20140185. 10.1098/rstb.2014.0185 26009764 PMC4455754

[B52] Riegel AC, Lupica CR (2004) Independent presynaptic and postsynaptic mechanisms regulate endocannabinoid signaling at multiple synapses in the ventral tegmental area. J Neurosci 24:11070–11078. 10.1523/JNEUROSCI.3695-04.2004 15590923 PMC4857882

[B53] Robinson BG, Cai X, Wang J, Bunzow JR, Williams JT, Kaeser PS (2019) RIM is essential for stimulated but not spontaneous somatodendritic dopamine release in the midbrain. Elife 8:e47972. 10.7554/eLife.47972 31486769 PMC6754207

[B54] Saez TMM, Fernandez Bessone I, Rodriguez MS, Alloatti M, Otero MG, Cromberg LE, Pozo Devoto VM, Oubiña G, Sosa L, Buffone MG, Gelman DM, Falzone TL (2020) Kinesin-1-mediated axonal transport of CB1 receptors is required for cannabinoid-dependent axonal growth and guidance. Development 147:dev184069. 10.1242/dev.184069 32265198 PMC7188441

[B55] Salvatore MF, McInnis TR, Cantu MA, Apple DM, Pruett BS (2019) Tyrosine hydroxylase inhibition in substantia nigra decreases movement frequency. Mol Neurobiol 56:2728–2740. 10.1007/s12035-018-1256-9 30056575 PMC6349536

[B56] Scheyer AF, Borsoi M, Pelissier-Alicot AL, Manzoni OJJ (2020) Perinatal THC exposure via lactation induces lasting alterations to social behavior and prefrontal cortex function in rats at adulthood. Neuropsychopharmacology 45:1826–1833. 10.1038/s41386-020-0716-x 32428929 PMC7608083

[B57] Schindelin J, Arganda-Carreras I, Frise E, Kaynig V, Longair M, Pietzsch T, Preibisch S, Rueden C, Saalfeld S, Schmid B, Tinevez JY, White DJ, Hartenstein V, Eliceiri K, Tomancak P, Cardona A (2012) Fiji: an open-source platform for biological-image analysis. Nat Methods 9:676–682. 10.1038/nmeth.2019 22743772 PMC3855844

[B58] Schneider M (2009) Cannabis use in pregnancy and early life and its consequences: animal models. Eur Arch Psychiatry Clin Neurosci 259:383–393. 10.1007/s00406-009-0026-0 19572160

[B59] Sgobio C, Wu J, Zheng W, Chen X, Pan J, Salinas AG, Davis MI, Lovinger DM, Cai H (2017) Aldehyde dehydrogenase 1-positive nigrostriatal dopaminergic fibers exhibit distinct projection pattern and dopamine release dynamics at mouse dorsal striatum. Sci Rep 7:5283. 28706191 10.1038/s41598-017-05598-1 PMC5509666

[B60] Shima Y, Sugino K, Hempel CM, Shima M, Taneja P, Bullis JB, Mehta S, Lois C, Nelson SB (2016) A Mammalian enhancer trap resource for discovering and manipulating neuronal cell types. Elife 5:e13503. 10.7554/eLife.13503 26999799 PMC4846381

[B61] Steiner H, Bonner TI, Zimmer AM, Kitai ST, Zimmer A (1999) Altered gene expression in striatal projection neurons in CB1 cannabinoid receptor knockout mice. Proc Natl Acad Sci U S A 96:5786–5790. 10.1073/pnas.96.10.5786 10318962 PMC21938

[B62] Stella N (2010) Cannabinoid and cannabinoid-like receptors in microglia, astrocytes and astrocytomas. Glia 58:1017–1030. 10.1002/glia.20983 20468046 PMC2919281

[B63] Tung LW, Lu GL, Lee YH, Yu L, Lee HJ, Leishman E, Bradshaw H, Hwang LL, Hung MS, Mackie K, Zimmer A, Chiou LC (2016) Orexins contribute to restraint stress-induced cocaine relapse by endocannabinoid-mediated disinhibition of dopaminergic neurons. Nat Commun 7:12199. 10.1038/ncomms12199 27448020 PMC4961842

[B64] Van Laere K, Casteels C, Dhollander I, Goffin K, Grachev I, Bormans G, Vandenberghe W (2010) Widespread decrease of type 1 cannabinoid receptor availability in Huntington disease in vivo. J Nucl Med 51:1413–1417. 10.2967/jnumed.110.077156 20720046

[B65] Witkovsky P, Patel JC, Lee CR, Rice ME (2009) Immunocytochemical identification of proteins involved in dopamine release from the somatodendritic compartment of nigral dopaminergic neurons. Neuroscience 164:488–496. 10.1016/j.neuroscience.2009.08.017 19682556 PMC2879289

[B66] Wu CS, Zhu J, Wager-Miller J, Wang S, O’Leary D, Monory K, Lutz B, Mackie K, Lu HC (2010) Requirement of cannabinoid CB(1) receptors in cortical pyramidal neurons for appropriate development of corticothalamic and thalamocortical projections. Eur J Neurosci 32:693–706. 10.1111/j.1460-9568.2010.07337.x 21050275 PMC2970673

[B67] Zeng BY, Dass B, Owen A, Rose S, Cannizzaro C, Tel BC, Jenner P (1999) Chronic L-DOPA treatment increases striatal cannabinoid CB1 receptor mRNA expression in 6-hydroxydopamine-lesioned rats. Neurosci Lett 276:71–74. 10.1016/S0304-3940(99)00762-4 10624794

[B68] Zhou FW, Jin Y, Matta SG, Xu M, Zhou FM (2009) An ultra-short dopamine pathway regulates basal ganglia output. J Neurosci 29:10424–10435. 10.1523/JNEUROSCI.4402-08.2009 19692618 PMC3596265

[B69] Zimmer A, Zimmer AM, Hohmann AG, Herkenham M, Bonner TI (1999) Increased mortality, hypoactivity, and hypoalgesia in cannabinoid CB1 receptor knockout mice. Proc Natl Acad Sci U S A 96:5780–5785. 10.1073/pnas.96.10.5780 10318961 PMC21937

