# Supplementary figures and images for: Cannabinoid Receptor 1 Is Required for Neurodevelopment of Striosome-Dendron Bouquets
Source: eNeuro. 2022 Apr 8;9(2):ENEURO.0318-21.2022. doi: 10.1523/ENEURO.0318-21.2022 (PMC9007419; doi:10.1523/ENEURO.0318-21.2022)

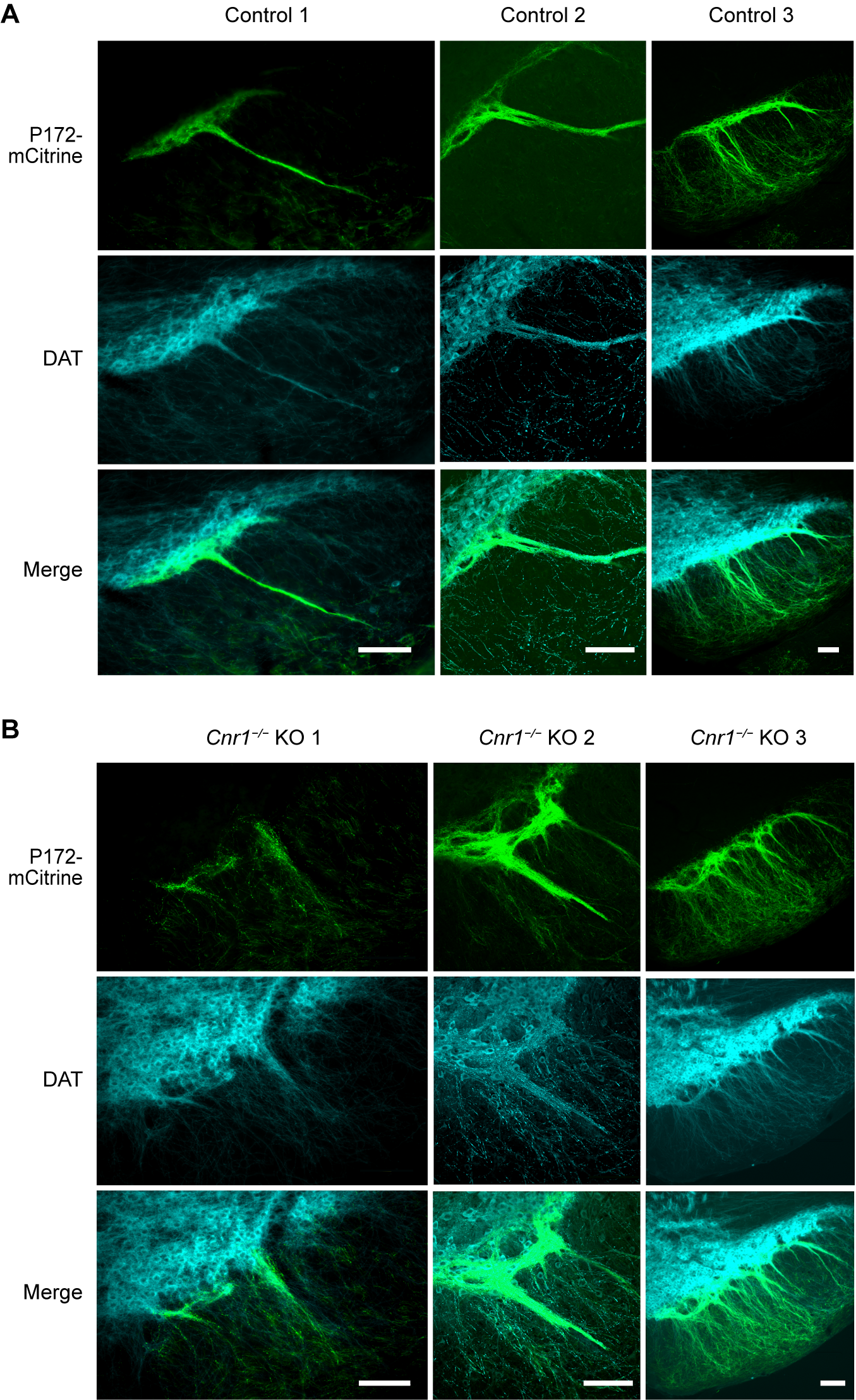

Supplement: Extended Data Figure 2-1 — Striosome-dendron bouquet samples in in the SN of controls (A) and Cnr1−/− KO (B) mice. Images of the SN from three mice of each genotype showing disorganized and loosely fasciculated striosomal axons (P172-mCitrine fluorescence in green) and dopaminergic dendrites (cyan, DAT immunolabeling in cyan). Coronal sections from the left hemisphere are shown. Scale bars: 100 μm. Download Figure 2-1, TIF file. [file enu-eN-NWR-0318-21-s04.tif]

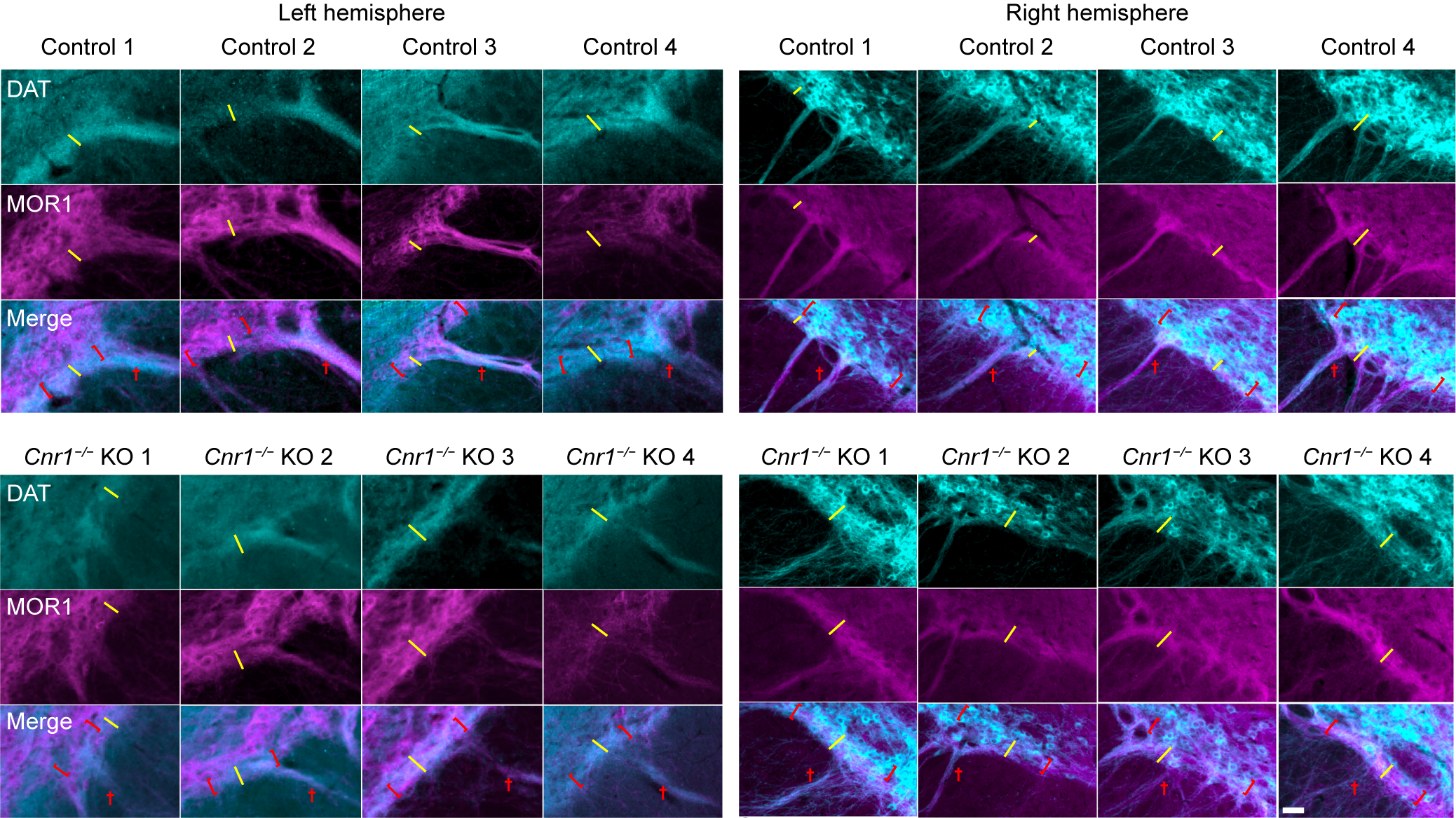

Supplement: Extended Data Figure 5-1 — A subset of images from coronal nigral sections used to measure anatomical features in adult Cnr1−/− KO mice and controls. The SNcv were defined for being immunopositive for both MOR1 and DAT and are delineated by red brackets in the merged image; dendrons are designated by a red cross. Measurements were taken along the white line (one line per section). N = 4 mice of each genotype, balanced for sex, n = 3 sections per hemisphere for each mouse. Scale bar: 10 μm (lower right panel and applies to all panels). Download Figure 5-1, TIF file. [file enu-eN-NWR-0318-21-s05.tif]

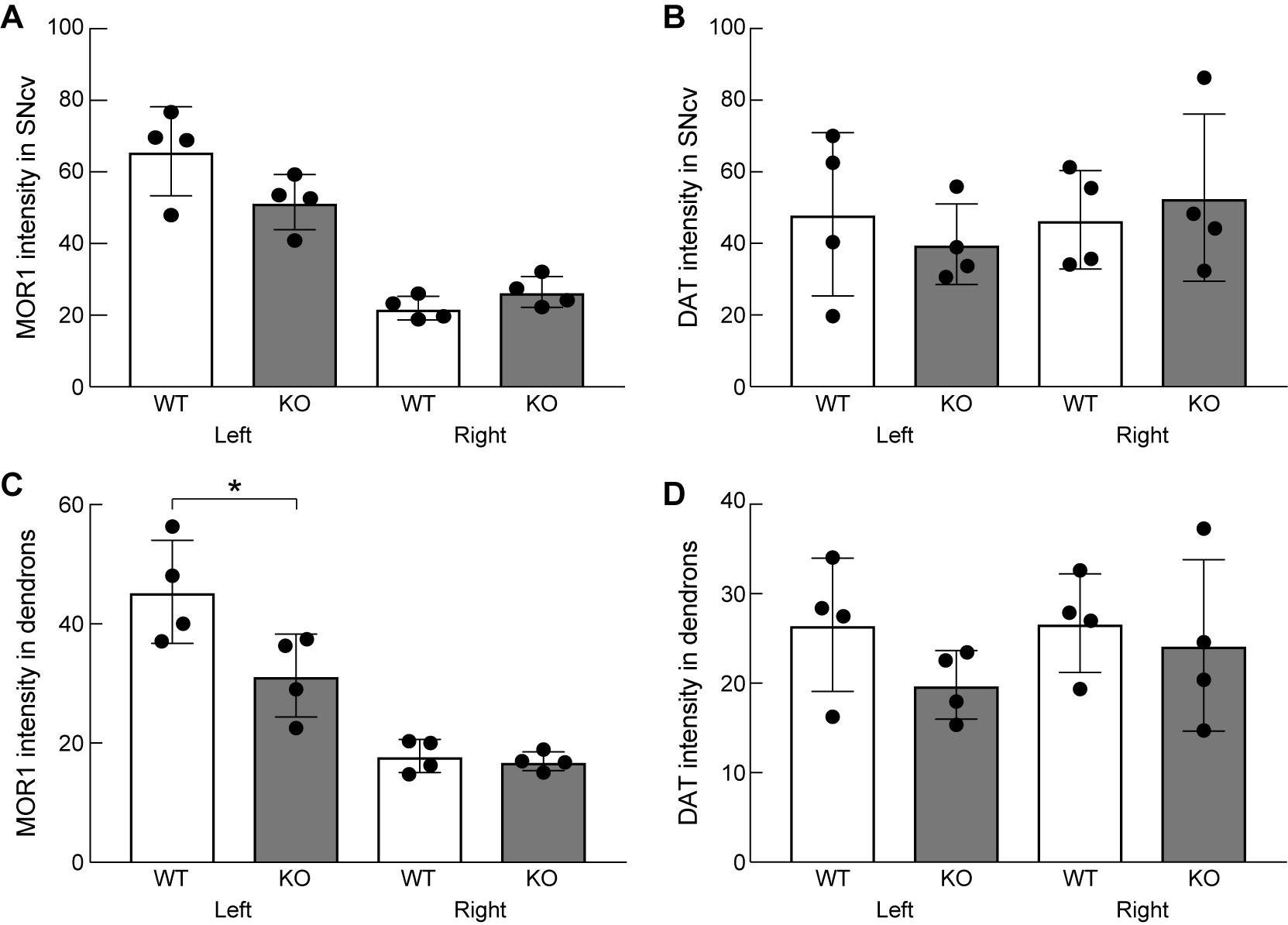

Supplement: Extended Data Figure 5-2 — Immunolabeling for MOR1 and DAT in nigral sections from Cnr1−/− KO mice and controls. There were few significant genotype differences in MOR1 (A, C) and DAT (B, D) immunolabeling (arbitrary units) of SNcv (A, B) or dendrons (C, D). Genotype comparisons were made only between same-side hemispheres because they were processed together but separately from the opposite hemisphere (p > 0.05 for comparisons between genotypes except for MOR1 immunointensity in left hemisphere dendrons for which *p = 0.046). Download Figure 5-2, TIF file. [file enu-eN-NWR-0318-21-s06.tif]

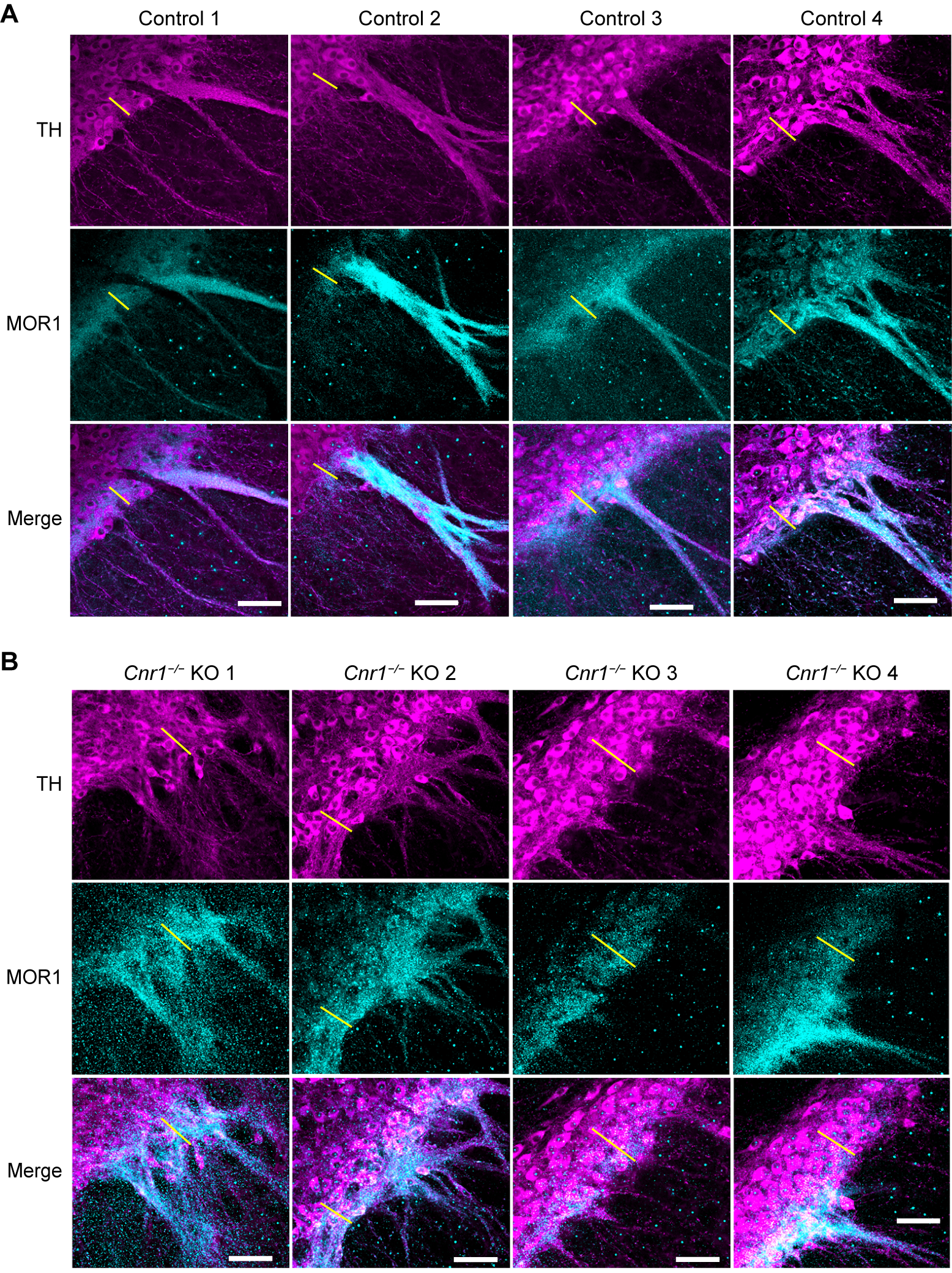

Supplement: Extended Data Figure 13-1 — Coronal sections through the left nigra of controls (A) and sibling Cnr1−/− KO (B) pups. Co-labeling for TH and MOR1 was used to measure the thickness of the SNcv, defined by double-labeled region and designated by yellow line. Scale bars: 50 μm (shown in the merged images panel). Download Figure 13-1, TIF file. [file enu-eN-NWR-0318-21-s07.tif]
